# Bacterial DNA methyltransferase: A key to the epigenetic world with lessons learned from proteobacteria

**DOI:** 10.3389/fmicb.2023.1129437

**Published:** 2023-03-22

**Authors:** Qun Gao, Shuwei Lu, Yuwei Wang, Longgui He, Mingshu Wang, Renyong Jia, Shun Chen, Dekang Zhu, Mafeng Liu, Xinxin Zhao, Qiao Yang, Ying Wu, Shaqiu Zhang, Juan Huang, Sai Mao, Xumin Ou, Di Sun, Bin Tian, Anchun Cheng

**Affiliations:** ^1^Research Center of Avian Diseases, College of Veterinary Medicine, Sichuan Agricultural University, Chengdu, Sichuan, China; ^2^Key Laboratory of Animal Disease and Human Health of Sichuan Province, Chengdu, Sichuan, China; ^3^Institute of Preventive Veterinary Medicine, Sichuan Agricultural University, Chengdu, Sichuan, China; ^4^Key Laboratory of Livestock and Poultry Provenance Disease Research in Mianyang, Sichuan, China

**Keywords:** DNA methyltransferase, DNA methylation, restriction-modification systems, orphan methyltransferase, phase variation, epigenetics

## Abstract

Epigenetics modulates expression levels of various important genes in both prokaryotes and eukaryotes. These epigenetic traits are heritable without any change in genetic DNA sequences. DNA methylation is a universal mechanism of epigenetic regulation in all kingdoms of life. In bacteria, DNA methylation is the main form of epigenetic regulation and plays important roles in affecting clinically relevant phenotypes, such as virulence, host colonization, sporulation, biofilm formation et al. In this review, we survey bacterial epigenomic studies and focus on the recent developments in the structure, function, and mechanism of several highly conserved bacterial DNA methylases. These methyltransferases are relatively common in bacteria and participate in the regulation of gene expression and chromosomal DNA replication and repair control. Recent advances in sequencing techniques capable of detecting methylation signals have enabled the characterization of genome-wide epigenetic regulation. With their involvement in critical cellular processes, these highly conserved DNA methyltransferases may emerge as promising targets for developing novel epigenetic inhibitors for biomedical applications.

## 1. Introduction

DNA methylation is a process that adds methyl groups to DNA nucleotides by enzymes known as DNA methyltransferase (Adhikari and Curtis, 2016). This process is involved in regulating a wide range of cellular processes, including epigenetic regulations in bacteria. Epigenetics is a change in gene expression that is heritable without a change in the DNA sequence itself. Unlike eukaryotes, which employ complex epigenetic regulation mechanisms, bacterial epigenetic control is primarily achieved through DNA methylation. It superimposes secondary information on a primary DNA sequence, adding additional directions to DNA transactions such as transcription, transposition, initiation of chromosome replication, and prevention of mutations by DNA repair (Marinus and Casadesús, 2009; Kumar et al., 2018; Estibariz et al., 2019; Tourancheau et al., 2021). DNA methylation is found throughout the prokaryotic kingdom, with S-adenosyl-L-methionine (SAM) as a common methyl group donor. However, DNA methyltransferases have been shown to be very diverse.

**Figure 1:**
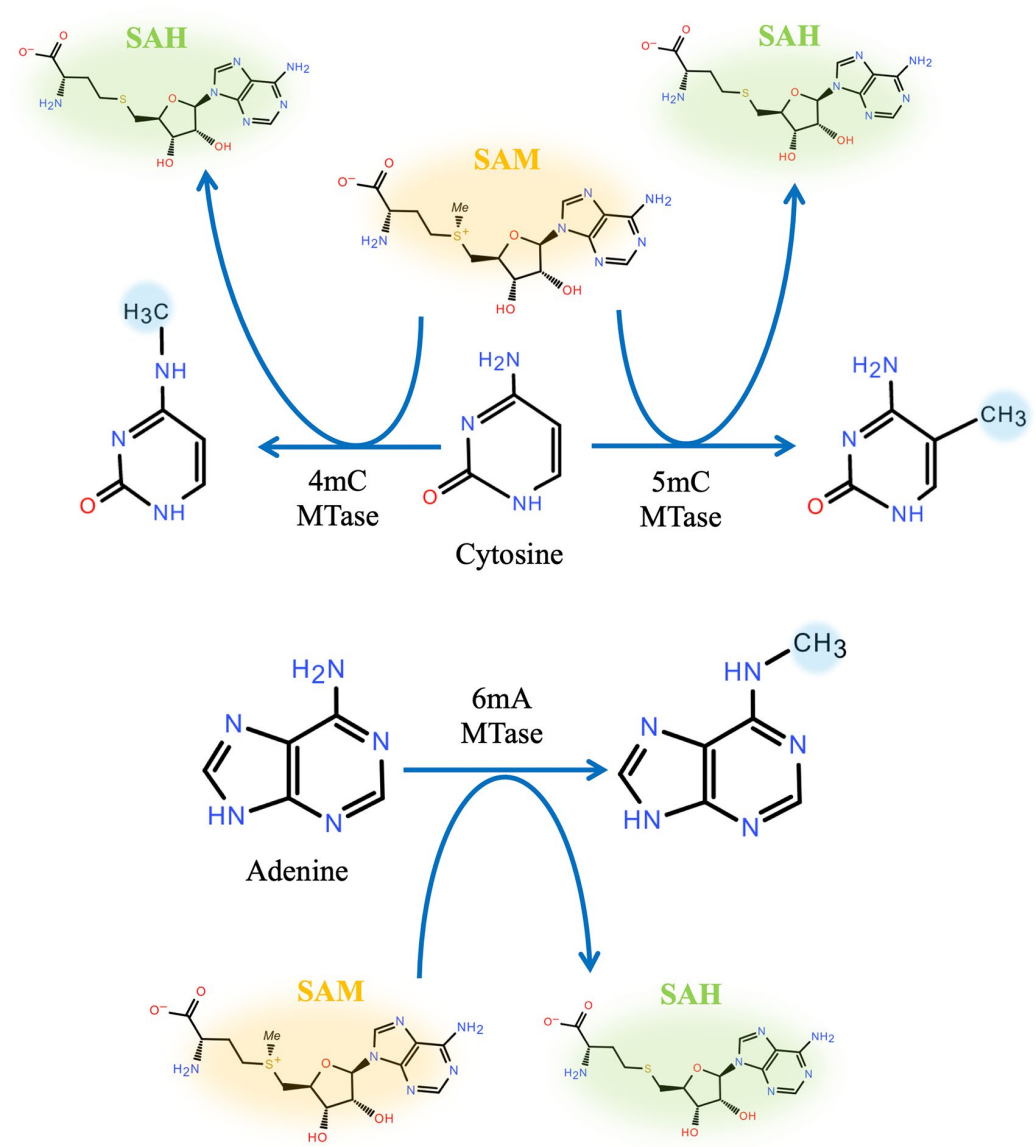
Position of DNA methylation. Cytosine methylation can be either endocyclic (C^5^) or exocyclic (N^4^). Adenine can be methylated at N^6^.

Based on the position to which the methyl group is transferred, DNA methyltransferases can be divided into two classes: exocyclic amino methyltransferases and endocyclic methyltransferases. Exocyclic amino methyltransferase transfers a methyl group to the N^4^ position of cytosine (N^4^-C) or the N^6^ position of adenine (N^6^-A), e.g., Dam and CcrM. Endocyclic methyltransferase methylates cytosine at the C^5^ position (C^5^-C), e.g., Dcm (Wion and Casadesús, 2006; Marinus and Casadesús, 2009; Kumar et al., 2018; Chen S. et al., 2022). Among these variants, C^5^-C is predominantly found in eukaryotes, whereas N^4^-C and N^6^-A are mainly found in bacteria, Figure 1.

There are two main categories of DNA methyltransferases: methyltransferases in Restriction-Modification systems (“R-M systems”) and “solitary” or “orphan” methyltransferases. The R-M systems constitute DNA methyltransferases (MTases) and associated restriction enzymes (REases) (Bickle and Krüger, 1993). In a majority of bacteria, the R-M systems function like an immune response, protecting their own DNA while degrading foreign DNA (Lees and Gladstone, 2015). Host DNA is methylated by a DNA methyltransferase which protects against digestion from the cognate restriction endonuclease, whereas foreign DNA, such as invading phage DNA, is unmethylated and degraded by the endonuclease (Bickle and Krüger, 1993). However, protecting the integrity of its own genomic DNA is not the sole purpose of R-M systems. Studies have found that MTases from R-M systems are also involved in regulating gene expression (Lees and Gladstone, 2015; Seib et al., 2020).

Solitary DNA methyltransferases, also known as orphan methyltransferases, were identified much later than the R-M systems and have no associated restriction endonucleases (Adhikari and Curtis, 2016). The best-characterized orphan MTases are Dam, Ccrm, and Dcm (Kahng and Shapiro, 2001; Løbner-Olesen et al., 2005; Militello et al., 2020). Studies have found that orphan MTases are involved in regulating important cellular processes, such as initiation of DNA replication, DNA repair, and gene regulation (Lu et al., 1994; Von Freiesleben et al., 1994; Van der Woude et al., 1996; Casadesús and Low, 2006; Wion and Casadesús, 2006).

Genes encoding DNA methyltransferases are widely present in the genomes of bacteria, indicating that DNA methyltransferases play an important role in bacteria. Distinct bacterial lineages and phenotypic heterogeneity are common in bacteria. The formation of bacterial subpopulations from the same lineage is often controlled by epigenetic mechanisms that generate inheritable phenotypic diversity without altering the DNA sequence (Casadesús and Low, 2013; Sánchez-Romero et al., 2015; Tan et al., 2016; de Ste Croix et al., 2017). However, how DNA methyltransferases are involved in many cellular processes remains unknown. Recent developments in single-molecule real-time (SMRT) sequencing and nanopore DNA sequencing technologies have made detecting methylated bases achievable. They undoubtedly provide us with an accessible tool for studying DNA methylation in bacteria and may open a new era in bacterial epigenomics by deciphering a wealth of information on bacterial genome methylation patterns and functional consequences (Clarke et al., 2009; Flusberg et al., 2010; Attar, 2016; Tourancheau et al., 2021; Chen J. et al., 2022). This review summarizes some of the properties of bacterial DNA methyltransferases and highlights recent developments in understanding the role of DNA methyltransferase in physiological processes, especially gene expression regulation in bacteria.

## 2. Two main DNA methylation systems

### 2.1. Restriction-modification systems

Genes encoding R-M systems are present on most bacterial and archaeal genomes. Their prevalence indicates how important R-M systems are to prokaryotes. The well-known function role of R-M systems is to protect the host cell from invading foreign DNAs (Murphy et al., 2013). Canonical R-M systems contain enzymes carrying out two activities: a restriction endonuclease, which binds to specific recognition sites and hydrolyzes DNA when the sequence is unmethylated, and a corresponding DNA methyltransferase, which methylates DNA on the same sites recognized by their cognate endonuclease. Some DNA methyltransferases, such as M.EcoGII, could methylate not only residues in DNA but also those in DNA:RNA-hybrid oligonucleotide duplexes (Murray et al., 2018).

Because of their ability to distinguish “self” from “non-self,” the R-M systems are considered to provide a primitive immune system (Vasu and Nagaraja, 2013). Consequently, R-M systems are thought to be essential for host colonization by pathogenic bacteria (Ershova et al., 2015).

R-M systems are classified into four main types based on factors such as the subunit composition of its REase and MTase complexes; ATP and cofactor requirements; recognition site structure; and DNA-cleavage site (Murphy et al., 2013; Ershova et al., 2015). Typical Type I R-M systems consist of three subunits: two modification subunits and one S-subunit. The S-subunit is encoded by *hsdS* as a specificity subunit, which can specifically bind to recognition sites. The two modification subunits are encoded by *hsdM* as methyltransferase (M) and by *hsdR* as restriction endonuclease (R). The MTase in Type I can modify both strands of its substrate DNA. There are two target recognition domains (TRD) in the S-subunit, and each interacts with half of the bipartite recognition site (Murray, 2000; Loenen et al., 2014). Functional restriction activity requires a pentamer comprised of R_2_M_2_S.

Type II R-M systems are the most prevalent and simplest. They generally function as two individual proteins. The REase cleaves the target DNA at defined positions within or close to its recognition site, and the MTase protects host DNA by methylation. They are also the best-investigated group of R-M systems because Type II REases are key enzymes in genetic engineering. Type III R-M systems comprise two subunits: Restriction (Res) and Modification (Mod) enzymes, encoded by the *res* and *mod* genes, respectively. Mod binds to and methylates substrate DNA, while Res functions as a DNA restriction endonuclease. Interestingly, Mod can function independently of Res, whereas Res has no activity without Mod (Dryden et al., 2001). Finally, Type IV R-M systems, distinctive from the other three types, hydrolyze modified DNA. Type IV systems have the methyltransferase and endonuclease activity combined in a single enzyme, which exclusively cleaves modified DNA (Vasu and Nagaraja, 2013).

In addition to their traditionally understood role, the R-M systems have been found to be involved in epigenetic regulation in some bacteria (Table 1). Several studies have reported that methyltransferases in Type III R-M systems have sequence features that are consistent with phase-variable expression (Srikhanta et al., 2005; Fox et al., 2007; Li and Zhang, 2019; Anton and Roberts, 2021). Phase variation is a strategy to generate phenotypic diversity in a bacterial population in the absence of selection. It involves reversible, high-frequency ON/OFF switching of gene expression. Phase variation is often mediated by the presence of highly mutagenic simple tandem DNA repeats, also known as simple sequence repeats (SSRs). The SSRs are often located either within the ORF of genes encoding variant proteins or in their promoter region. Recent research has identified phase variably expressed DNA methyltransferases that act as epigenetic regulators in several pathogenic bacteria (Tan et al., 2016; Lyko, 2018; Han et al., 2022). Many virulence factor genes in bacteria display phase-variable expression, such as pili (Srikhanta et al., 2017), iron-binding proteins (Tauseef et al., 2011), lipopolysaccharide biosynthesis genes (Lüneberg et al., 1998), and outer-membrane proteins (Owen et al., 1996; Green et al., 2019). Phase variation results in genetically and phenotypically diverse populations, which is important in pathogenesis as it provides rapid adaptation to changes brought by the host environment and immune responses (Atack et al., 2018). The DNA methyltransferases that are involved in phase variation themselves are often subject to phase-variable expression (Atack et al., 2015; Tan et al., 2016). It is conceivable that a methyltransferase that is involved in phase variation can cause even more variation possibilities once the methyltransferase itself goes through a phase variation. The numerical possibility of phase variation multiplies by an amplitude when the variable methyltransferase itself is phase varied. The existence of phase-variable methyltransferase raises the possible roles for phase-variable R-M systems in pathogenesis. Using the flagellin A (*flaA*) gene in *Helicobacter pylori* as a model, we know that the ModH5 (a Type III MTase) modulates *flaA* promoter activity in a methylation-dependent manner. The phase-variable switching of ModH5 expression plays a role in regulating *Helicobacter pylori* phenotypes (Srikhanta et al., 2017).

**Table 1:** Physiological role of bacterial DNA methyltransferases.

Type	Name	Bacteria	Target sequence	Methylation position	DNA methylation involved in physiological processes	
					Regulation target	Other physiological processes
DNA methyltransferases of R-M systems	ModM2	*Moraxella catarrhalis*	5′-GARAC-3′	N^6^-adenines	Pathogenicity (Blakeway et al., 2014)	Protecting the integrity of the bacterial genome (Estibariz et al., 2019)
	ModA	*Haemophilus influenzae*	5′-CCGAA-3′ 5′-CGAG-3′ 5′-ACAGC-3′ 5′-CCTGA-3′ 5′-CCTAC-3′		Phenotype and escape from the killing of phagocytes (Atack et al., 2015)	
		*Neisseria meningitidis & Neisseria gonorrhoeae*	5′-CGYAG-3′ 5′-ACACC-3′ 5′-AGAAA-3′		Virulence (Srikhanta et al., 2009)	
	M.HpyIII	*Helicobacter pylori*	5′-GCGC-3′	C^5^-cytosines	Growth, viability, shape (Estibariz et al., 2019)	
	M2.HpyAII	*Helicobacter pylori*	5′-TCTTC-3′	N^4^-cytosines	Virulence & ribosomal assembly (Kumar et al., 2018)	
“solitary” or “orphan” methyltransferases	Dam	*Escherichia coli*	5′-GATC-3′	N^6^-adenines	*pap*—Lrp (Bacterial colonization) (Van der Woude et al., 1996); sci1—Fur (Biofilm formation) (Brunet et al., 2011); Flu—OxyR (Biofilm formation) (van der Woude and Henderson, 2008)	IS10 transposition —transposase (Roberts et al., 1985); Chromosome replication —DnaA, SeqA (Campbell and Kleckner, 1990; Waldminghaus and Skarstad, 2009)；Mismatch repair—MutH (Li, 2008); Prevention in constitutive stable DNA replication(cSDR) (Raghunathan et al., 2019); Chromosome segregation (Barras and Marinus, 1989)
	CcrM	*Caulobacter crescentus*	5′-GANTC-3′		*ctrA*—GcrA (Regulation of the cell cycle) (Adhikari and Curtis, 2016)	Unknown yet
	Dcm	*Escherichia coli*	5′-CCAGG-3′ 5′-CCTGG-3′	C^5^-cytosines	Regulating the stress response factor RPOS and increasing the expression of SUGE, affecting bacterial resistance (Kahramanoglou et al., 2012; Militello et al., 2014)	Inducing a high mutation rate in bacteria (Bandaru et al., 1996)

### 2.2. “Solitary” or “orphan” methyltransferase

In addition to the R-M systems, some methyltransferases exist with no association with any restriction enzymes and are designated as “solitary” or “orphan” methylases (Casadesús and Low, 2006). MTases from R-M systems are distinct from orphan MTases. MTases from R-M systems can cleave unmethylated foreign DNA, whereas orphan MTases cannot. In general, MTases in R-M systems are poorly conserved, whereas orphan MTases are as conserved within a genus as any average gene (Seshasayee et al., 2012). Despite the differences, orphan MTases may have been derived from the R-M systems through loss of function in the REase of an R-M system. An R-M system in which the restriction endonuclease lost its activity but its cognate DNA methyltransferase retained its activity is functionally equivalent to orphan methyltransferases (Casadesús and Low, 2006). Some studies have found that orphan methyltransferases may have originated through horizontal gene transfer, with only the MTase part of an R-M system being transferred (Oliveira et al., 2014). Orphan MTases are present in diverse bacterial and archaeal phyla and show motif specificities and methylation patterns that are consistent with functions in gene regulation and DNA replication (Blow et al., 2016). In addition, there is a theory that orphan MTases might have evolved from R-M systems to fight parasitism caused by selfish or rogue R-M systems. However, there is currently a lack of evidence to support this theory (Murphy et al., 2013).

There are three well-studied conserved orphan methyltransferases: Dam, Ccrm, and Dcm, as shown in Table 1. Among them, the Dam of γ *Proteobacteria* and the CcrM of *Caulobacter crescentus* are the most widely studied (Chen et al., 2014). Dam is an orphan MTase first found in *Escherichia coli* and is involved in methyl-directed mismatch DNA repair and regulation of chromosomal replication (Marinus and Morris, 1973; Urig et al., 2002; Marinus and Casadesús, 2009). Its homologs are present in various enteric bacteria, including *Yersinia* spp., *Vibrio cholerae*, *Salmonella* app., *Haemophilus influenzae*, and other genera (Barras and Marinus, 1989; Torreblanca and Casadesús, 1996; Zaleski and Piekarowicz, 2004; Giacomodonato et al., 2009; Banas et al., 2011). Dam methylation can influence the expression of virulence factors and, thereby, the pathogenicity of some bacteria (Pucciarelli et al., 2002; Watson et al., 2004; Ershova et al., 2015). Modified live attenuated *S. enterica* serovar Typhimurium that harbor loss-of-function mutations in *dam* are capable of eliciting protection against a diversity of *Salmonella* and are well-tolerated when applied as modified live vaccines in poultry, mice, calves, and sheep (Heithoff et al., 2015). Although Dam is not essential for most bacteria, it is necessary for the survival of *V. cholerae* (Torreblanca and Casadesús, 1996; Robinson et al., 2005; Taylor et al., 2005; Casadesús and Low, 2006; Val et al., 2014).

Dam is a 32 kDa monomeric protein that catalyzes the transfer of the methyl group from AdoMet to the N^6^ position of the adenine residue in 5′GATC3′ sequences. Dam has similar efficiency for both hemi- and unmethylated templates and has been shown to be involved in chromosome replication and segregation, DNA mismatch repair, regulation of transposition events, phase variation, and bacterial conjugation processes (Sánchez-Romero et al., 2015). In fact, all DNA methyltransferases can either methylate a half-methylated DNA strand or simultaneously methylate two unmethylated strands of DNA (Urig et al., 2002). Notably, Dam is a processive enzyme and could continuously methylate adenine in 5′GATC3′ sites, with about 55 sites being methylated per binding event (Brooks et al., 1983; Urig et al., 2002; Bheemanaik et al., 2006; Marinus and Casadesús, 2009). That explains the disproportionally low ratio of Dam to 5′GATC3′ sites.

The cell cycle-regulated DNA MTase family (CcrM) constitutes a second important group of orphan methyltransferase (Skerker and Laub, 2004; Kozdon et al., 2013; Ershova et al., 2015). It plays an essential role in the life cycle of *C. crescentus* and is highly conserved in all α *Proteobacteria*, except for *Rickettsiales* and *Magnetococcales* (Brilli et al., 2010; Mouammine and Collier, 2018). In *Caulobacter*, CcrM is an essential cell component and plays a crucial role in cell cycle regulation (Casadesús and Low, 2006). However, it is not present for the entire life cycle and is only expressed right before cell division. Evidence suggests that CcrM participates in the cell-cycle regulation of *C. crescentus* through regulating the expression of cell-division genes (Zweiger et al., 1994; Stephens et al., 1996; Reisenauer and Shapiro, 2002; Fioravanti et al., 2013). Interestingly, the culture conditions seem to determine the dependence on CcrM methyltransferase in *Caulobacter* (Gonzalez and Collier, 2013; Adhikari and Curtis, 2016). In some α *Proteobacteria*, such as *Brucella abortus*, *C. crescentus*, and *Agrobacterium tumefaciens*, CcrM methyltransferase is indispensable (Stephens et al., 1996; Robertson et al., 2000). However, it is not vital for cell growth in other α *Proteobacteria*, like in *Brecundimonas subvibrioides* (Robertson et al., 2000; Curtis and Brun, 2014).

CcrM is a functional monomer but may form a dimer at physiologic concentration (Shier et al., 2001; Skerker and Laub, 2004; Kozdon et al., 2013; Casadesús, 2016; Horton et al., 2019). Unlike Dam, CcrM has a distinct preference for hemimethylated DNA substrates (Bheemanaik et al., 2006; Albu et al., 2012). It binds to DNA, and only one 5′GANTC3′ (N represents any base) site can be methylated and modified before the enzyme detaches from the DNA (Marinus and Casadesús, 2009).

Dcm is another orphan MTase that is mainly found in enterobacteria such as *E. coli*. Dcm is a 51KD protein that methylates cytosine in a position that is rarely modified in bacteria but commonly in eukaryotes. As a C^5^-cytosine methyltransferase, Dcm can methylate the second cytosine in 5′CCAGG 3′ and 5′CCTGG 3′ sites (Militello et al., 2014). Methylated 5′CAG3′ sequences are mutational hotspots. After deamination, 5-methylcytosine is thymine and is not removed by the uracil-N-glycosylase, so GC to AT mutations are common at 5′ CCAGG 3′ sites. Dcm participates in several cellular processes, but it has been shown that Dcm is not necessary for the survival of *E. coli* (Baba et al., 2008).

## 3. Structure, function, and mechanism of DNA methyltransferases

### 3.1. Structure

Currently, there are 27 bacterial DNA MTases that have been crystallized with three-dimensional structures determined.^1^ Their target sequences are summarized in Table 2 (Roberts et al., 2015). Most of these DNA MTases belong to Type I or II R-M systems, and structures for orphan methyltransferases and Type III R-M systems are relatively rare. In general, MTases are bilobed structures folded into two domains: one is the catalytically active region responsible for the transfer of methyl groups, and the other is a smaller region responsible for recognizing methylation sites on DNA (Bergerat et al., 1991; Bheemanaik et al., 2006), Figure 2. There is a common structural core in the larger catalytic region, consisting of a six-stranded parallel β-sheet with a seventh strand inserted in an antiparallel fashion between the fifth and sixth strands (Bheemanaik et al., 2006). This larger catalytic region can be divided into two subdomains. One of the subdomains creates a S-adenosylmethionine (AdoMet) binding site, and the other subdomain is the binding site for the extrahelical target base. The small domains are very diverse in amino acid sequence, size and structure, because of selection for their DNA binding specificity (Bheemanaik et al., 2006). In all cases, the substrate to be methylated is bound or expected to bind in a pocket adjacent to the AdoMet binding site.

**Table 2:** Summary of DNA methyltransferases whose structure has been determined.

Site	Name	Target sequence	Type	Ref
m6A	M.BthVORF4518P	Unknown	I	Park et al. (2012)
	M.VvuYORF266P			
	M.Sth18311ORF711P			
	M.MmaGORF429P			
	DpnM	5′-GATC-3′		Tran et al. (1998)
	M.EcoKI	5′-AACNNNNNGTGC-3′		Kennaway et al. (2009)
	M.EcoR124I	5′-GAANNNNNRTCG-3′		Taylor et al. (2012)
	M.TteMI	5′-CACNNNNNNNTGC-3′		Liu et al. (2017)
	LlaBIII*	5′-GGCTNA-3′		Kulkarni et al. (2016)
	LlaGI*	5′-CRTCNAG-3′		
	M1.HpyAVI	5′-GAGG-3′	II	Ma et al. (2016)
	BpuSI	5′-GGGAC-3′		Shen et al. (2011)
	M.MboIIA	5′-GAAGA-3′		Osipiuk et al. (2003)
	MmeI	5′-TCCRAC-3′		Callahan et al. (2016)
	M.RsrI	5′-GAATTC-3′		Scavetta et al. (2000)
	M.TaqI	5′-TCGA-3′		Goedecke et al. (2001)
	Dam	5′-GATC-3′	Orphan methyltransferase	Horton et al. (2015)
	CcrM	5′-GANTC-3′		Horton et al. (2019)
	M.HpyAXI	5′-GCAG-3′	III	Narayanan et al. (2020)
	Ecop15I	5′-CAGCAG-3′		Gupta et al. (2015)
m5C	M.EcoKO157ORF1953P	Unknown	II	
	M.HaeIII	5′-GGCC-3′		Reinisch et al. (1995)
	M.HhaI	5′-GCGC-3′		Cheng et al. (1993)
	M.MpeI	5′-CG-3′	Orphan methyltransferase	Albert et al. (2018)
	M.SflTDcmP	Unknown		
m4C	M.PvuII*	5′-CAGCTG-3′	II	Gong et al. (1997)
	TTHA0409	Unknown		Morita et al. (2008)

**Figure 2:**
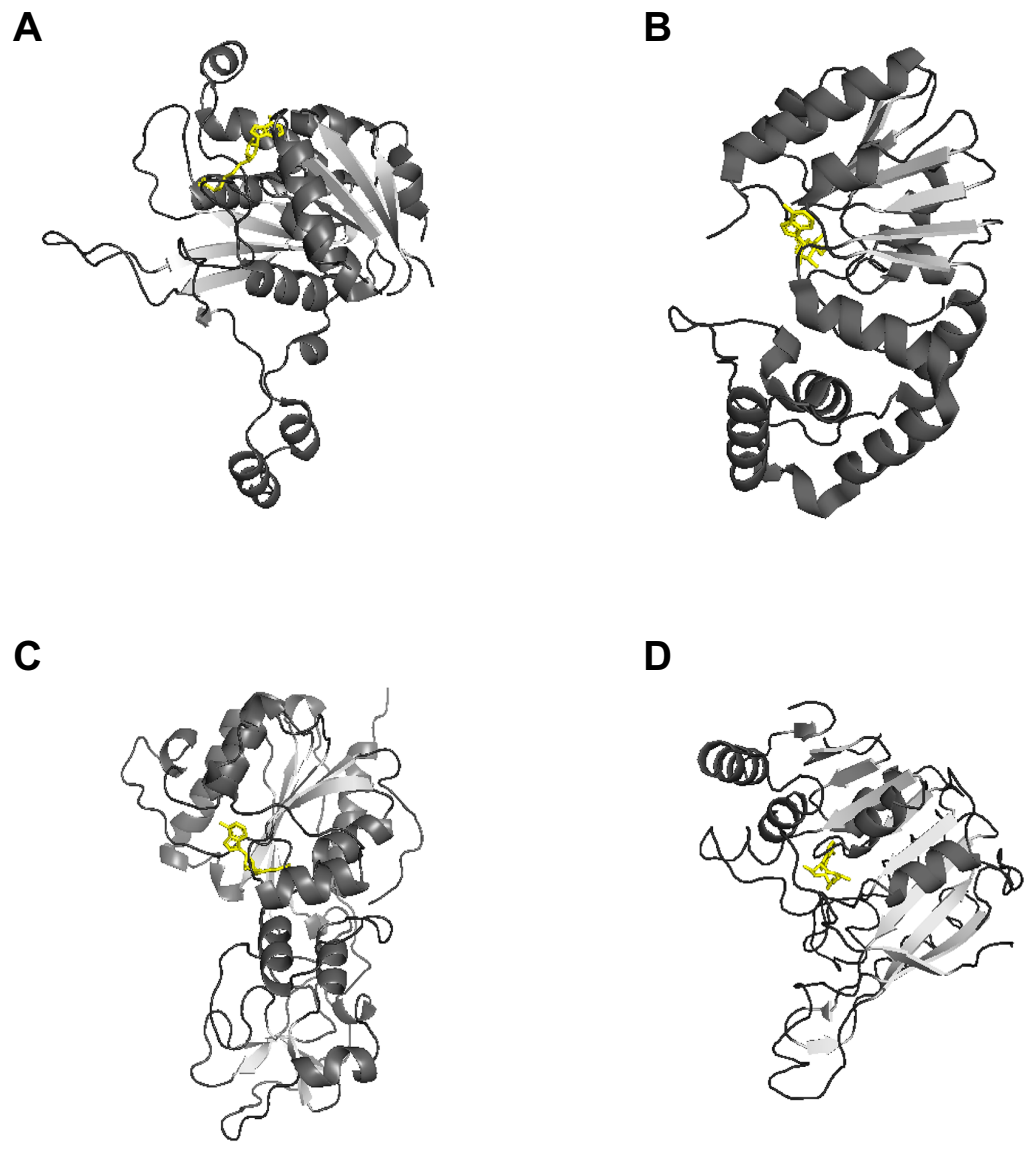
Representative three-dimensional structure of a DNA methyltransferase. AdoMet molecules are shown in yellow. These representative MTases all have a two-domain structure. **(A)** Crystal structure of RsrI with AdoMet (PDB code 1NW7). RsrI is a β-class N^6^-adenine MTase that recognizes the palindromic duplex DNA sequence GA **A** TTC and methylates the second adenine on each strand (Thomas et al., 2003). **(B)** Crystal structure of Dam with AdoMet (PDB code 2ORE). It contains two domains: a seven-stranded catalytic domain that harbors the binding site for AdoHcy and a DNA binding domain consisting of a five-helix bundle and a β-hairpin loop that is conserved in the family of GATC-related MTase orthologs (Liebert et al., 2007). **(C)** Crystal structure of HhaI with AdoMet (PDB code 2HMY). It is an endocyclic MTase that methylates the cytosine at the C^5^ position (O'Gara et al., 1999). The structure of the binary complex of M. HhaI with AdoMet (PDB code 1HMY) was the first structure of any AdoMet-dependent MTase reported (Hong and Cheng, 2016). **(D)** The crystal structure of Pvull with AdoMet (PDB code 1BOO). The main feature of the common fold is a seven-stranded β-sheet (6↓7↑5↓4↓1↓2↓3↓) formed by five parallel β-strands and antiparallel β-hairpin (shown as arrows). The AdoMet binding site is located at the carboxyl ends of strands β1 and β2, and the active site is formed by the carboxyl ends of strands β4 and β5 and the amino end of the strand β7. (Woodcock et al., 2020).

### 3.2. Motif

The primary sequences of MTases share a set of conserved motifs (I-X) and a variable target-site- recognition domain located near the C-terminus (Lauster et al., 1989; Pósfai et al., 1989). Together, they are responsible for three basic functions: (i) AdoMet binding, (ii) sequence-specific DNA binding, and (iii) catalysis of methyl group transfer. Briefly, motif I accommodates the methionine moiety of AdoMet, which is conserved among all AdoMet-dependent enzymes as a main binding region with AdoMet. In fact, all DNA methyltransferases that use S-adenosylmethionine as methyl donor are remarkably conserved in motif I.

Motifs II and III are also involved in AdoMet binding, but are less conserved than motif I. Several conserved charged residues in motifs I–III have been shown to have substantial effects on AdoMet binding (Ahmad and Rao, 1996). In motif I, substituting certain glycine residues with aspartic acid or arginine residues abolishes AdoMet binding. However, charged residues are not the only determining factor in AdoMet binding. Hydrophobic side chains in motifs I–III have been shown to stabilize AdoMet binding as well (Roth et al., 1998).

Motif IV, which is critical for catalysis, is also known as the DPPY motif because it contains a consensus sequence of (S, N/D)PP(Y/W/F) (Bheemanaik et al., 2006; Horton et al., 2006, 2019). The prolyl dipeptide on Motif IV is considered to play an important role in the transfer of methyl group to the target base (Smith et al., 1990; Cheng and Roberts, 2001). This sequence constitutes an active pocket that can accommodate the target base. DNA methyltransferase can modify the target base when it enters the active pocket through base flipping (Bheemanaik et al., 2006). Aromatic side chains in motif V have been found to interact with AdoMet (Schluckebier et al., 1995). Motifs VI, VII, and VIII form a DNA-binding cleft, whereas motifs I, IV, and X form a binding pocket for AdoMet’s methionine moiety (Cheng and Roberts, 2001).

### 3.3. Methyltransferase-DNA interactions

In general, the mechanism by which DNA methyltransferase recognizes methylation modification sites mainly depends on the surface between the enzyme and the major groove of the substrate double-stranded DNA. The enzyme interacts with deoxyribose and phosphate on the backbone through Van der Waals forces (Roberts and Cheng, 1998; Cheng and Roberts, 2001). Although there are a large number of conserved sequences and homologous structures between different DNA methyltransferases, the interactions between DNA recognition sites and methyltransferase show many variations. The DNA backbone is often severely distorted when a methyltransferase binds to the recognition site.

Methyltransferase Dam contains two domains: a seven-stranded catalytic domain (residues 1–56 and 145–270) harboring the binding sites for AdoMet/S-Adenosyl-L-homocysteine (AdoHcy or SAH) and a DNA binding domain (residues 57–144) consisting of a five-helix bundle and a β-hairpin loop (residues 118–139) (Horton et al., 2006). Dam uses a mechanism called base-flipping to methylate the target adenine. Side chains of the residues in the binding site directly interact with the phosphate groups of DNA backbone. Dam has four conserved residues (R95, N126, N132, and R137) that interact with three consecutive phosphate groups flanking the fourth GATC base-pair of the non-target strand. The methylation target, the adenine of the second base-pair in GATC, is flipped out from the DNA helix. The specific interactions with the remaining bases of the site occur in the DNA major groove. The N-terminal K9 is responsible for recognizing the guanidine of the first base-pair. Contacts to the non-target strand in the second half of the GATC site are established by R124 with the fourth base pair and by L122 and P134 to the third base-pair. The aromatic ring of Y119 intercalates into the DNA between the second and third base-pairs, which is essential for base-flipping to occur (Horton et al., 2006), Figure 3A.

**Figure 3:**
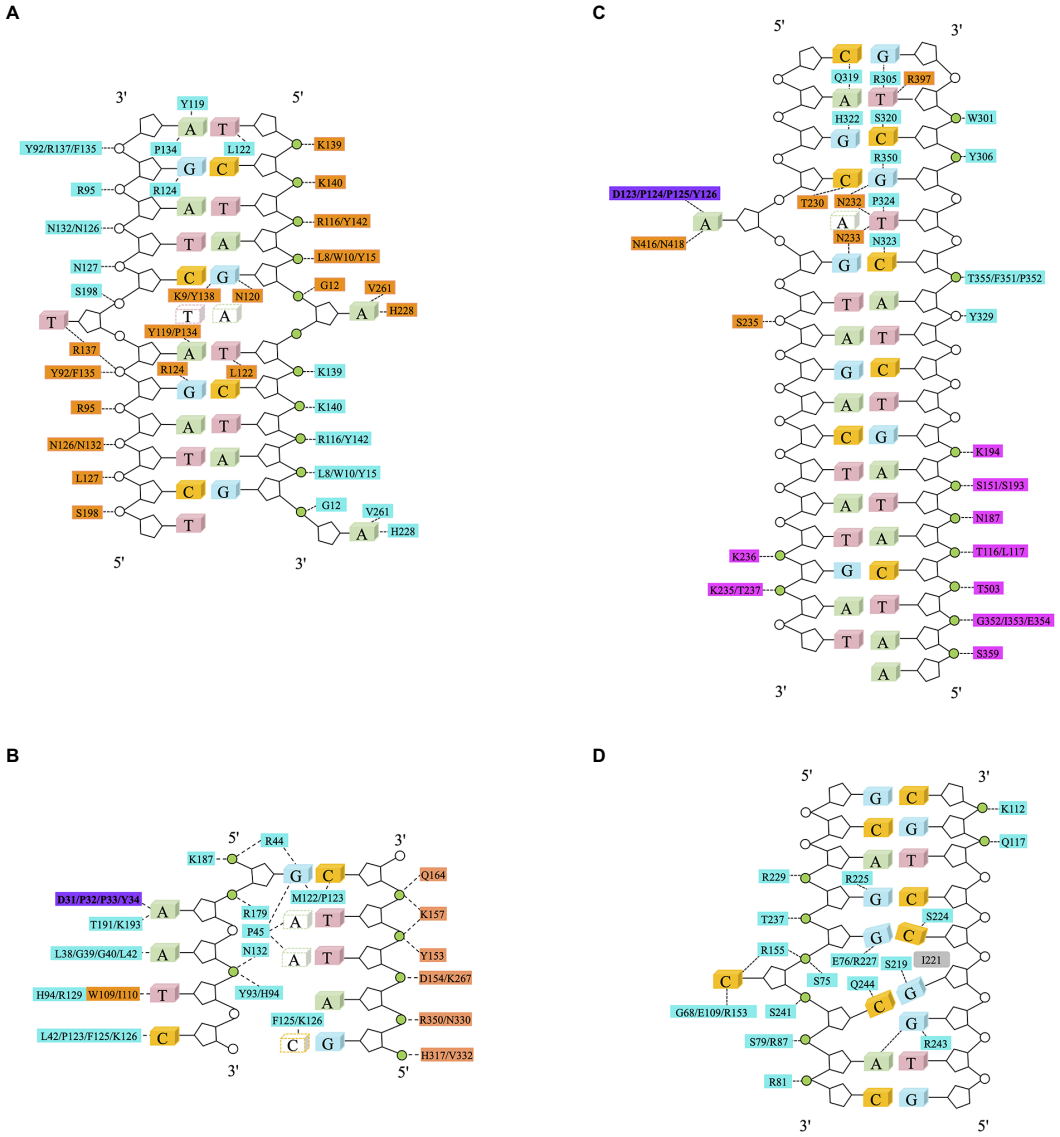
Schematic diagram of DNA methyltransferase-DNA contacts. Amino acids from the MTase domain of molecule A (in cyan) and the C-terminal domain of molecule B (in orange) collaborate for DNA binding. The purple rectangle represents an active pocket of DNA methyltransferase, the green circle represents phosphoric acid that interacts with amino acids. Non-target bases are in white, dashed line represents Van der Waals or hydrogen bonds. **(A)** Schematic of Dam and the – DNA interactions. Twenty out of 22 phosphate groups of the DNA interact with Dam residues (A in cyan and B in orange). The only two phosphate groups that are not involved in the interaction between DNA and Dam are the 5′ phosphate groups of the two Thy of the central GATC site, which are the phosphate groups missing from the joint GATC site (Horton et al., 2006). **(B)** Schematic of CcrM-DNA interaction. The two domains of CcrM contribute unevenly to DNA binding. Molecule A (in cyan) makes most of the contacts to the target strand (in green) and catalyzes methyl transfer, whereas the C-terminal domain of molecule B (in orange) is solely responsible for the binding of the non-target strand (in white) (Horton et al., 2019). **(C)** Schematic of the EcoP15I-DNA interaction. Residues from EcoP15I which are involved in direct interaction with DNA are colored as follows: ModA in cyan, ModB in orange, Res in magenta. The adenine in the dotted rectangle rotates ~180° out of DNA helix and enters the ModB catalytic cleft (Gupta et al., 2015). **(D)** Schematic of the M.HhaI-DNA interaction. The intercalating side chain of I221 is shown in gray (Didovyk and Verdine, 2012).

The dimeric CcrM methyltransferase uses a different mechanism to recognize the 5′ GAN^6^ATTC 3′ methylation site (Woodcock et al., 2017; Reich et al., 2018). Four loops of CcrM are involved in recognition of methylation sites. However, almost all the phosphates on the non-target base chain come into contact with Loop-2B (residues 31–61), which recognizes the first three bases of the target sequence. The P45 in Loop-2B is inserted between the second and third base pairs. Loop-45 (residues 119–133) can be inserted into the DNA double helix from a minor groove, where P125 and F126 recognize the fifth base in the target site to provide interactions with the two pyrimidines, T^4^ and C^5^. The shorter Loop-3C (residues 92–94) and Loop-6E (residues 172–194) supply additional interactions for thymine T^4^ (Tyr93 and His94), target adenine A^2^ (Thr191 and Lys193), and the DNA backbone phosphate groups flanking guanine G1 (Arg179 and Lys187) (Horton et al., 2019), Figure 3B.

EcoP15I belongs to the type III R-M family. EcoP15I, like CcrM, also causes the backbone of its target DNA fragment to twist after binding (Gupta et al., 2015). EcoP15I binding target 5′ GANTC 3′ sequence (N represents any base). EcoP15I consists of two methylation (Mod) and one (or two) restriction (Res) subunits, resulting in a Mod2Res1 or Mod2Res2 complex. The Mod subunits are responsible for DNA recognition and methylation, whereas the Res subunits are responsible for ATP hydrolysis and cleavage. Cleavage only occurs if two recognition sites (5′CAGCAG3′) are in an inverted-repeat orientation, arranged either as “head-to-head” or “tail-to-tail” (Gupta et al., 2015), Figure 3C.

C^5^-cytosine methyltransferases catalyze cytosine methylation through intermediates in which the DNA is drastically remodeled. The target cytosine is usually buried in a DNA double helix, which hinders the catalytic reaction. In order to carry out the catalytic reaction, a distortion in the DNA occurs, and the target cytosine residue extruded from the DNA helix and plunged into the active site pocket of the enzyme (Sankpal and Rao, 2002). This base flipping was found in all members of the monomeric type II bacterial methyltransferases, like M.HhaI and M.HaeIII (Matthews et al., 2016). The targeting mechanism is particularly intriguing in the case of M.HaeIII, a bacterial C^5^-cytosine methyltransferase that not only extrudes the substrate cytosine but also induces frameshifted base pairing and the formation of a large gap in the duplex DNA recognition site (Didovyk and Verdine, 2012). The target recognition region (TRD) of M.HaeIII contacts most nucleotides in the recognition site directly, thereby stabilizing their internal helical conformation. Then, residue Ile-221 is inserted between the second and third base pair of the recognition sequence (5′GGCC3′), weakening the base stacking force between them and resulting in the extrusion of the target cytosine. Finally, with concomitant abandonment of pairing for the G and C, underwinding (caused by negative supercoiling) of the DNA double helix located near the target site causes the target base to be flipped out of the DNA double helix, Figure 3D.

Similarly, in Dam, the aromatic ring of tyrosine at 119 can insert between the second and third base pairs of 5′GATC3′, which is considered to be a necessary condition for base flipping (Horton et al., 2006). It is worth noting that the angle of the base flip is different for different MTases. In Dam and CcrM, the target base flips about 90°, while in M.HhaI and EcoP15I, the target base flips about 180° (Gupta et al., 2015). In addition, all four nucleotides in the target site of CcrM can be flipped to different degrees at the same time (Horton et al., 2019). The mechanism needs further investigation.

## 4. Epigenetic regulation of gene transcription

DNA methylation in the promoter or regulatory regions can be used to regulate transcription (Casadesús and Low, 2006; Wion and Casadesús, 2006). One of the mechanisms involves competition for DNA binding. DNA MTases will not be able to bind to nascent DNA strands if they are pre-occupied by DNA-binding proteins, which will maintain their unmethylated state (Van der Woude et al., 1996; Casadesús and Low, 2006). However, DNA-binding proteins cannot bind to the DNA if the binding site is methylated. Therefore, when the sites of methylation overlap or are adjacent to the binding sites of DNA-binding proteins, there is competition for DNA binding. DNA-binding proteins can thus change the activity of DNA MTases, which, in turn, affects DNA methylation (Kot et al., 2020).

It has been established that DNA methylation influences the expression of bacterial genes, which, in turn, regulates cell lifecycle and virulence (Seib et al., 2020). Regulation of gene expression obviously influences the ability of the bacteria to adapt to and survive in their environment (Casadesús and Low, 2013). Dam and CcrM MTase are two examples for which there is a relatively clear understanding of their role in epigenetic regulation (Adhikari and Curtis, 2016). DNA methylation can also regulate gene expression after transcription, but the mechanism is still unclear (Campellone et al., 2007; López-Garrido and Casadesús, 2010). For example, Dam express level can cause changes in the composition of O-antigen in *Yersinia enterocolitica*, but the mRNA level of the O-antigen gene cluster did not change. It is not clear whether it was mRNA being modified or other mechanisms. This process is still called epigenetic regulation and is often found in phase variation (Casadesús and Low, 2013).

### 4.1. Phase variation *via* the restriction modification systems

**Figure 4:**
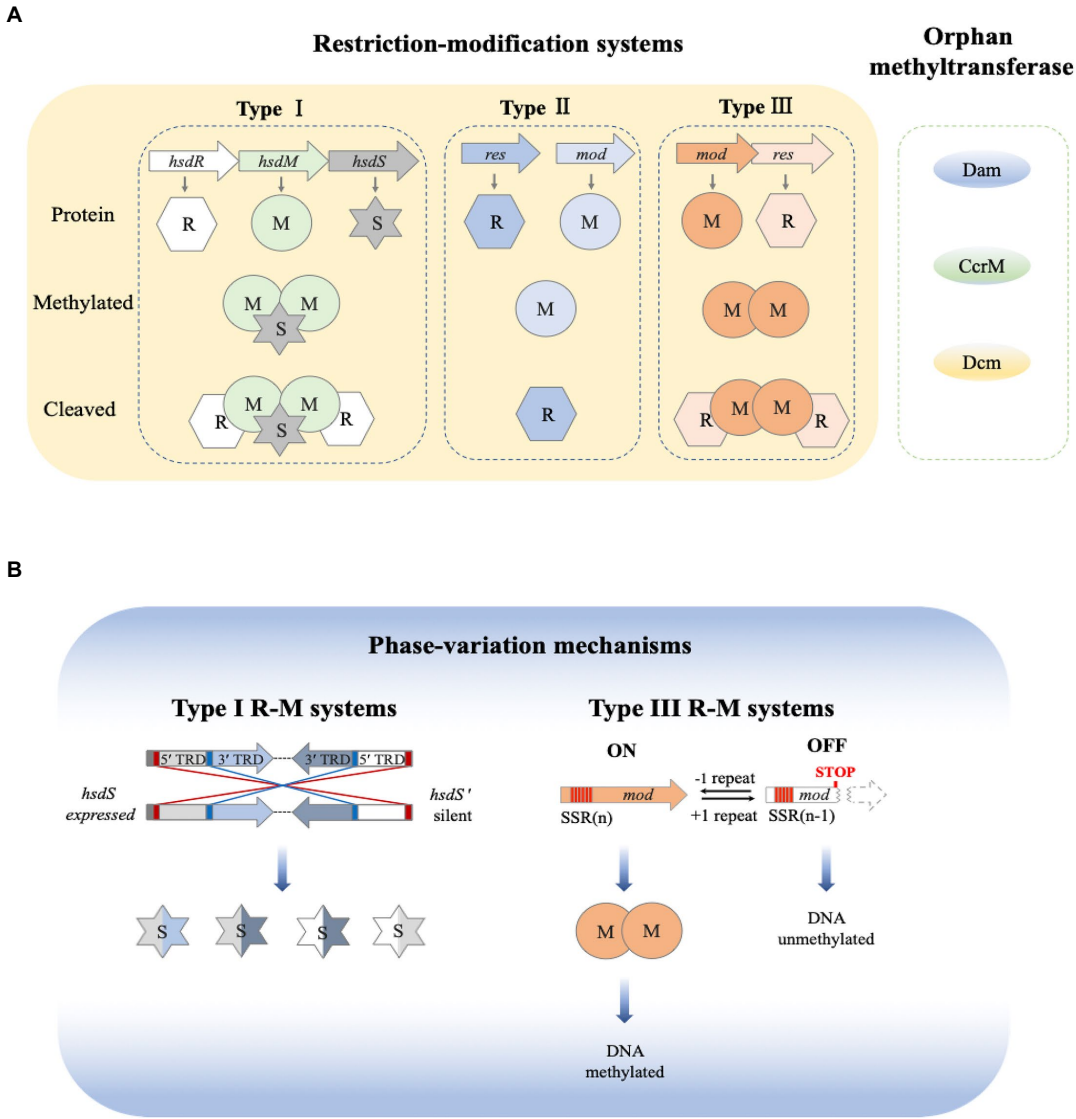
Overview of bacterial DNA methylation and phase variation. **(A)** Bacterial restriction-modification systems. There are three main types of restriction-methylation (R-M) systems and orphan methyltransferases. Type I R-M system consists of three components, which are encoded by *hsdR*, *hsdM* and *hsdS*, respectively. DNA methylation is mediated by a trimeric M2S complex, whereas DNA is cleaved by a pentameric R2M2S complex. Type II R-M systems are encoded by two individual genes. A single Mod subunit mediates the DNA methylation, whereas the DNA cleavage is mediated by one or two REase subunits. Type III R-M systems use two Mod (M2) subunits for DNA methylation, and R2M2 complexes for DNA cleavage. **(B)** Phase variation. Phase variation of Type I R-M system is mediated *via* inverted repeats (red at 5′ end, blue in center) to make recombination between expressed (*hsdS*) and silent (*hsdS′*) specificity genes. Each *hsdS* gene contains two target recognition domains (TRDs). Phase variation of type III R-M systems is achieved *via* slipped strand mispairing (SSM) of simple sequence repeats (SSR, in red) in the open-reading frame of the *mod* genes. By losing a repeat unit, the variation in the open reading frame shifts from expression of a functional Mod (Mod ON) to transcriptional termination (Mod OFF) (Seib et al., 2020).

Over the past two decades, R-M systems have been found not only to protect the integrity of the genome but also to participate in gene expression regulation (Seib et al., 2011; Blakeway et al., 2014; Kwiatek et al., 2015; Anjum et al., 2016; Tan et al., 2016; Srikhanta et al., 2017). Most R-M systems involved in the regulation of gene expression belong to type I and III, with a few belonging to type II, Figure 4A.

Phase variation, the reversible generation of variants of surface antigens, is a survival strategy that is frequently found in pathogenic bacteria (Wion and Casadesús, 2006). Genes that can undergo phase variation and change the expression of other genes are called phase-variable regulons (Tan et al., 2016). Phase-variable regulons can influence methylation patterns of the bacterial genome, which may further change the expression of other genes (Loenen et al., 2014). Phase-variable regulons can produce proteins with different specificities or directly switch gene expression through a phase-switching mechanism and then change the expression of other genes through epigenetic mechanisms. In fact, DNA methylation can regulate gene expression without changing gene sequence, thereby promoting phenotypic heterogeneity in bacterial populations and the formation of bacterial lineages (Cota et al., 2015; García-Pastor et al., 2018), Figure 5. It is worth noting that the coding genes of these R-M systems contain simple sequence repeats (SSR) that are easy to distinguish. These simple repetitive sequences can be a repetitive nucleotide or an inverted repetitive sequence (inverted sequences, IS) (Atack et al., 2018). Pathogens that have adapted to a host can change the number of simple repeats in the open reading frame or shuffle the inverted repeats to generate phase variation (Dupont et al., 2009; Atack et al., 2018). For example, in *H. pylori*, some DNA methyltransferase genes encoding type II R-M systems contain SSRs that are related to bacterial colonization and pathogenicity (Lin et al., 2001; Ando et al., 2010; Gauntlett et al., 2014).

**Figure 5:**
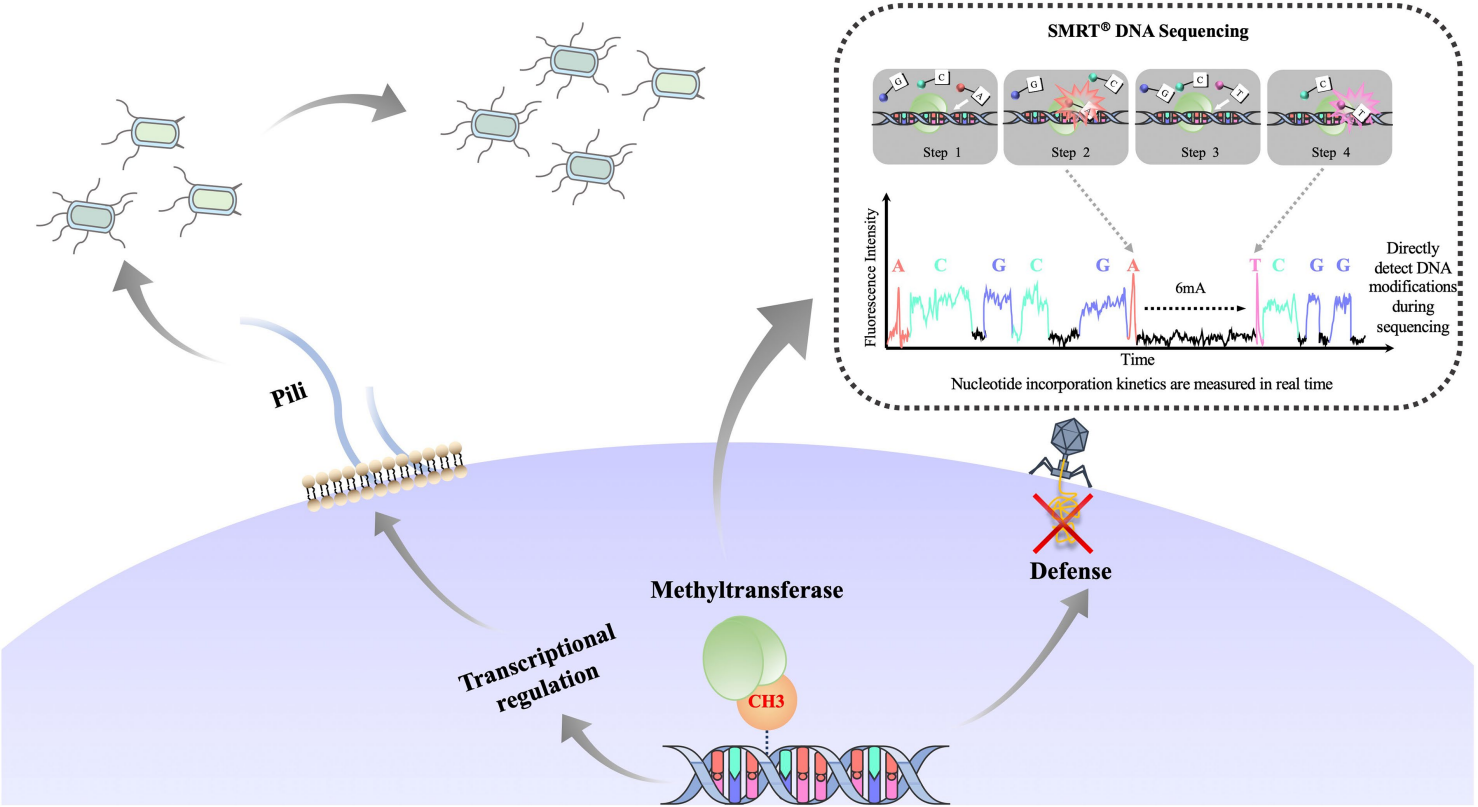
Overview of the function of bacterial DNA methylation and formation of bacterial phenotypically heterogeneous subgroups. DNA methyltransferase changes the expression *pap* operon by epigenetic regulation and forms phenotypically heterogeneous populations (long pili vs. short pili). DNA methylation defense mechanism can be used to protect the host genome from invasion by foreign DNA. Recent development in single molecule real-time (SMRT^®^) sequencing technology enables real-time sequencing of a large library of DNA fragments without PCR (dashed line inset; Attar, 2016; Chen J. et al., 2022). SMRT sequencing measures polymerase kinetics during the sequencing process, and thus detects DNA modification.

There are two different kinds of phase variation that have been observed in bacterial R-M systems. Most Type I R-M systems have been shown to carry out phase variation *via* homologous recombination, Figure 4B. Type I systems are encoded by *hsdR*, *hsdM*, *hsdS*, and 4.3% of *hsdR*, 2% of *hsdM*, and 7.9% of *hsdS* contain simple repetitive sequences which can regulate phase variation (Seib et al., 2020). Regulation of Type I systems can either increase or decrease the number of simple repeats on the coding genes in *hsdM* and *hsdS*. Phase variation can also be regulated through the recombination of the inverted repeats in *hsdS* to generate phase mutations. The number of simple repeats can regulate the expression of *hsdM*, which leads to the ON or OFF state of DNA MTase. The recombination of inverted repeats or the change in the number of simple repeats in the open-reading frame of *hsdS* leads to the production of HsdS with different specificities. Ultimately, the site at which methylation occurs changes (Manso et al., 2014).

In *H. influenzae*, the open reading frame of *hsdM*, which encodes the type I phase variation regulator, contains simple repeats of a 5′GACGA3′ sequence. Changing these repeats can regulate the expression of *hsdM* and the sensitivity of *H. influenzae* to phage infection (Zaleski and Piekarowicz, 2004; Atack et al., 2018). In *Neisseria gonorrhoeae*, *hsdS*, which encodes the Type I regulator of phase variation, contains two open-reading frames, *hsdSgoAV1* and *hsdSgoAV2*. There are multiple G residues at the 3′ end of *hsdSgoAV1* (polyG tract). Both *hsdSgoAV1* and *hsdSgoAV2* are expressed when *hsdSgoAV1* contains seven G residues at its 3′ end, whereas only *hsdSgoAV1* is expressed when there are six G residues. Therefore, the methylation and/or restriction sites can undergo phase variation (Adamczyk-Poplawska et al., 2011).

Another example is the Type I phase variation regulator SpnD39III in *Pneumococcus*, which contains an *hsdS* with two target-recognition regions (TRD) and two inverted repeats (IRs) of SpnD39III. The TRD and IR sequences can be shuffled, resulting in six different versions of *hsdS* (SpnIII39A-F) with different specificities (Manso et al., 2014). Expression of genes involved in capsule synthesis is down-regulated if SpnIII39B is produced. *Pneumococcus* with SpnIII39A has changes in the expression of genes involved in stress response and nutrient acquisition. However, no significant changes in gene expression have been detected with SpnIII39C or D (Manso et al., 2014).

In *Campylobacter jejuni*, there is a type II R-M system, Cj0031, containing polyG that could participate in regulating other genes. *Campylobacter jejuni* regulates the ON or OFF expression of Cj0031 by changing the number of G residues. This regulation can directly lead to the expression or absence of DNA methyltransferase in the system, which affects the methylation status of 5′CCYGA3′ sites on its genomic DNA. Some studies have found that when cj0031 is deleted or shut off by phase variation, the expression or absence of genes related to adhesion and invasion of host cells, such as *capA*, *cadF*, and *flpAde*, are down-regulated, whereas *peb1A*, a gene encoding periplasmic-binding proteins associated with ABC transporters, is up-regulated (Anjum et al., 2016).

In *H. influenzae*, there are multiple *modA* alleles encoding DNA methyltransferases that are involved in phase variation. These alleles include *modA1*, *modA2*, *modA4*, *modA5*, *modA9*, and *modA10* (Atack et al., 2015). These alleles contain some simple repetitive sequences, and their expression can be adjusted to ON or OFF (Srikhanta et al., 2005; Atack et al., 2015), Figure 4B. For example, the *modA1* open-reading frame contains several 5′AGCC3′ SSRs. The number of these repeats determines whether the expression of *modA1* is ON or OFF. The *modA1* expression level can affect the expression levels of sixteen other genes (Srikhanta et al., 2005). The bacteria can form a robust biofilm when *modA2* is expressed. The level of high molecular protein (HMP) in the cell decreases when the expression of *modA4* is turned off, which enhances escape from macrophages (Atack et al., 2015).

In *H. pylori*, there are 19 *mod* alleles encoding regulators of Type III phase variation (Srikhanta et al., 2010, 2017; Atack et al., 2018). Applying single molecule real-time (SMRT^®^) sequencing technology and methylome analysis (Figure 5 inset), it has been found that there is a 5′GACC3′ ModH5 methylation site in the promoter of *flaA*, which encodes the main component of the flagellar filament (Srikhanta et al., 2017). It has been suggested that methylation at the promoter region of *flaA* could directly regulate its expression. Similarly, a regulator of Type III phase variation is also reported in pathogenic bacteria *Neisseria gonorrheae* (Srikhanta et al., 2009), *Neisseria meningitidis*, *Mannella hemolyticus* (Srikhanta et al., 2010), and *Moraxella catarrhalis* (Blakeway et al., 2018). SSRs in the open reading frame of *mod* have been found to produce different regulators of Type III phase variation that affect the expression of other genes.

### 4.2. Gene regulation by Dam and CcrM

The Dam-regulated *pap* operon is a classic example of regulation by positive feedback (Van der Woude et al., 1996). Pap expression is regulated in a complex manner that involves PapB, PapI, Lrp (Leucine-responsive regulatory protein), and Dam. There are five promoters (P1–P5) in the regulatory region of the *pap* operon (Løbner-Olesen et al., 1992; Marinus and Casadesús, 2009). There are two main sets of Lrp binding sites, with each set containing a Dam methylation modification site 5′GATC3′. The methylation sites are named as GATC-I and GATC-II (van der Woude et al., 1992). Once Lrp binds to GATC-II, it prevents Dam from methylating GATC-II, resulting in a non-methylated state and blocking RNA polymerase σ70 from binding. In the meantime, GATC-I without Lrp binding is in a methylated state and the transcription of the *pap* operon is turned off (Pap-Off state). Conversely, when Lrp binds to GATC-I, resulting its being in an unmethylated state and GATC-II being in a methylated state, the transcription of the *pap* operon is turned on (Pap-On state) (Nou et al., 1995), Figure 6. It has been shown that the binding of PapI to Lrp *in vitro* reduces the affinity of Lrp for the first set of binding sites by half but increases the affinity of Lrp to the second set of binding sites by four times (Nou et al., 1995). Thus, when PapI expression reaches a certain level, it promotes the transfer of Lrp to the second set of binding sites, shifting from a Pap-On to a Pap-Off state. It has been reported that the frequency of is about 100 times higher than Pap-Off to Pap-On shifting (Blyn et al., 1990). On top of Lrp binding, the entire regulatory region of the *pap* operon can be bound by H-NS (a histone-like nucleoid structuring protein) (White-Ziegler et al., 1998), phosphorylated CpxR (CpxR-P) (Hernday et al., 2004), and RimJ (White-Ziegler et al., 2002) to affect the methylation status of the region.

**Figure 6:**
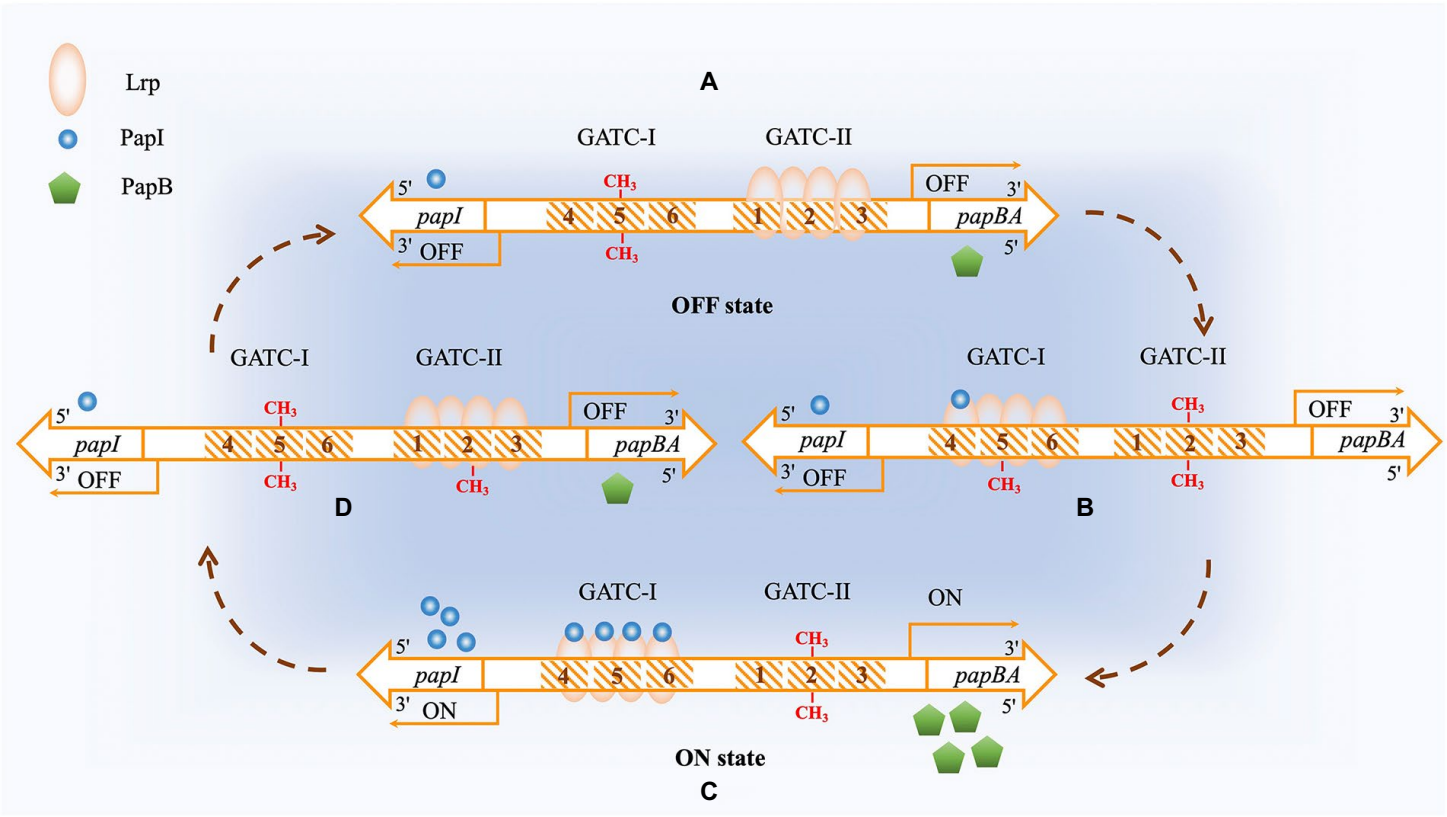
Dam methyltransferase is involved in the regulation of expression of the *pap* operon. The PapI protein is shown as blue circles, and the PapB protein is shown as green pentagons. The Lrp protein is a tetramer which is shown as an orange oval. **(A)** OFF state. The Lrp protein interacts with its sites 1–3. The GATC sequence in the fifth Lrp-binding site is methylated on both strands by Dam. The concentration of PapI protein is low. **(B)** After DNA replication, 4–6 Lrp binding sites are hemimethylated. Lrp binding with PapI increases its affinity to binding sites 4–6 that contain the hemimethylated GATC sequence. The interaction of Lrp with binding sites 4–6 protects the fifth Lrp binding site from Dam methylation but makes the second Lrp binding site available for methylation. The methylation of the second Lrp binding site decreases the affinity of the PapI/Lrp complex to binding sites 1–3 and increases the probability of binding to other sites. **(C)** ON state. The PapI/Lrp complex interacts with binding sites 4–6 and activates the transcription of the *pap* operon, expressing PapB protein. Increased PapB expression causes positive feedback of PapI protein expression. **(D)** More PapI protein results in increased binding of the PapI/Lrp complex to sites 1–3 after DNA replication, resulting in increased methylation at the fifth Lrp binding site, which inactivates the expression of the *pap* operon. The PapI concentration then decreases, completing the circle.

Another example of gene regulation is *opvAB*, which is a cytoplasmic membrane protein gene that can shorten the length of the lipopolysaccharide O-antigen of *Salmonella enterica* (Cota et al., 2012). The expression of *opvAB* is turned on when the length of the lipopolysaccharide O antigen needs to become shorter in order to escape infection by bacterial phages, which attach to the lipopolysaccharide O antigen. However, shortened lipopolysaccharide O antigen can reduce the ability to infect its host (Cota et al., 2015). In response to those needs, the expression of *opvAB* can be turned off in the absence of phage to restore the length of the O antigen.

It is worth noting that the genomes of *E. coli* and other γ-*Proteobacteria* have more 5′GATC3′ sequences than expected by chance (Hénaut et al., 1996; Sobetzko et al., 2016). This strongly suggests that Dam methyltransferase may be involved, in regulating transcription of many other genes. Some studies have found that in *E. coli*, *dam* mutations could affect the expression of many genes related to aerobic respiration, stress and SOS response, and amino acid and nucleotide metabolism (Oshima et al., 2002; Adhikari and Curtis, 2016). Studies have shown that Dam methyltransferase is involved in regulating transcription of *sci*1 (Brunet et al., 2011), *flu* (van der Woude and Henderson, 2008), *gtr* (Broadbent et al., 2010; Sánchez-Romero and Casadesús, 2020) and *std* (García-Pastor et al., 2019) in *E. coli* and *carA*, *dgoR*, *holA*, *nanA*, *ssaN*, *STM1290* and *STM3276* (Sánchez-Romero and Casadesús, 2020) in *S. enterica*.

However, there is no obvious relationship between Dam-involved gene transcription regulation and the presence of 5′GATC3′ sequences in the promoter of the regulated gene (Horton et al., 2015). Although the expression of many genes changes in *dam* mutants, only a few of those genes contain a 5′GATC3′ sequence in their promoters (Seshasayee, 2007). Several factors must be considered. In addition to 5′GATC3′, Dam methyltransferase can also react with 5′GTYTA3′/5′TARAC3′. The regulatory regions of the *pap*, *foo* and *clp* genes contain these non-5′GATC3′ sites (Horton et al., 2015). After the deletion of *dam* in enterohemorrhagic *E. coli* (EHEC), the expression of the Tir protein and the virulence protein EspFU increased, but their mRNA levels did not change (Campellone et al., 2007). In *Yersinia enterocolitica*, overexpression of Dam methyltransferase changed the composition of O-antigen, but the mRNA level of the O-antigen gene cluster did not change (Fälker et al., 2007). Similarly, the involvement of Dam methyltransferase in regulating the expression of *hild* seem to be post-transcriptional (López-Garrido and Casadesús, 2010). Therefore, regulation of gene expression involving Dam can occur after transcription, a process which probably does not involve 5′GATC3′ sites on the genomic DNA. The mechanism of post-transcription regulation by Dam or other DNA methyltransferases is still unclear.

The genome of *C. crescentus* contains 4542 CcrM methylation modification sites (5′GANTC3′), of which 23% are located inside a cluster of genes (Kozdon et al., 2013). In addition, it has been reported that when CcrM in *C. crescentus* is deleted or overexpressed, 10% of the genes are misexpressed, and expression of 380 genes changes significantly (Gonzalez et al., 2014). The genes that were significantly affected by the level of CcrM contain at least one 5′GANTC3′ site in their respective promoters. It has been shown that CcrM participates in regulating the transcription of *flaY*, *podJ*, *ftsN*, *mipZ* and *ctrA* in *C. crescentus* (Adhikari and Curtis, 2016). The *ctrA* gene contains two promoters, P1 and P2, each containing a 5′GANTC3′ methylation modification site (Domian et al., 1999). However, the 5′GANTC3′ site in P2 does not seem to be involved in the regulation of *ctrA* (Reisenauer and Shapiro, 2002). CcrM regulates the gene *ctrA* through a corresponding transcriptional regulator GcrA (Reisenauer and Shapiro, 2002). During DNA replication, the replication fork passes through P1, which makes P1 hemimethylated. GcrA binds to the hemimethylated P1 and activates promoter P1, which transcribes *ctrA*, resulting in the production of a small quantity of CtrA protein (Holtzendorff et al., 2004). CtrA binds to a specific site on the promoter P2, leading to a rapid increase in the level of CtrA in the cell (Domian et al., 1999). The vast majority of CtrA comes from P2 promoter, which demonstrates that the P1 promoter is only active in the hemimethylated state (Reisenauer and Shapiro, 2002).

The timing and level of CtrA expression are critical to the cell cycle of *C. crescentus*. They coordinate the growth and division of pre-divisional cells. CtrA also regulates the expression of more than 90 genes (Laub et al., 2002). One of the 90 genes that CtrA regulates is *ccrM*. The surge of CtrA leads to an increase of the CcrM level in the cell. The promoter of *ccrM* contains two 5′GANTC3′ methylation sites. When chromosome DNA is replicated, two hemimethylated 5′GANTC3′ methylation sites are formed in the pre-divional cell, resulting the surge of CtrA and the increased expression of *ccrM* gene (Stephens et al., 1996; Reisenauer et al., 1999).

## 5. DNA methyltransferase and chromosome replication

**Figure 7:**
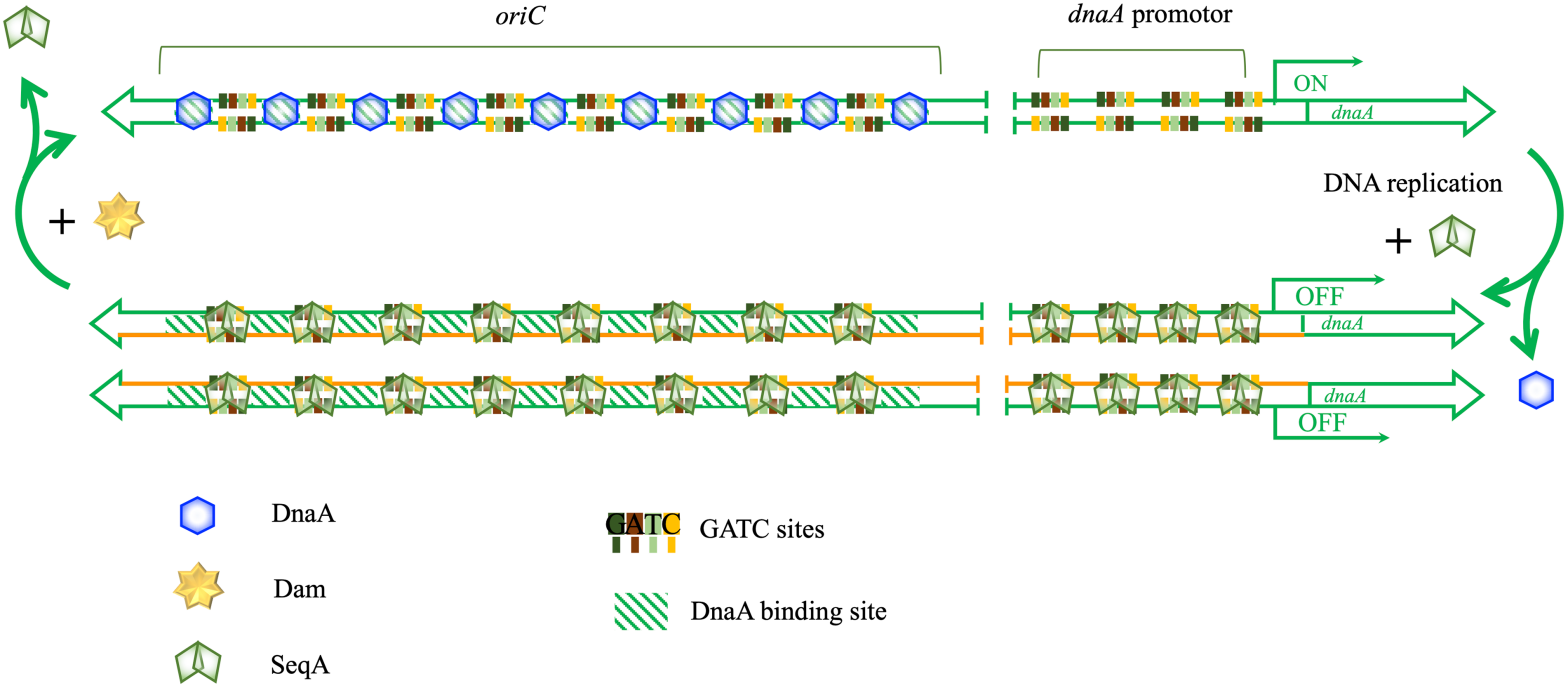
Dam methyltransferase is involved in the initiation of DNA replication. There are multiple GATC sites in *oriC* and *dnaA* promotors of *E*. *coli*. DnaA-binding sites are shown with green diagonal stripe rectangles. DnaA binds to its binding sites in *oriC* and starts melting *oriC* to initiate replication. Then, SeqA binds to the newly hemimethylated DNA and excludes DnaA from binding, preventing re-initiation. In addition, SeqA binds to the hemimethylated *dnaA* promotor to prevent the expression of *dnaA*.

*Escherichia coli* chromosome replication is controlled primarily at the level of initiation, and the frequency of initiation determines the rate of cell division (Bogan and Helmstetter, 1997). One important element in the regulation of timing of initiation is the methylation status of the nascent DNA strand. *Escherichia coli* uses three different mechanisms to control the initiation of DNA replication: controlling the expression of *dnaA* (Campbell and Kleckner, 1990), sequestration of *oriC*, the replication origin, by SeqA protein (Bogan and Helmstetter, 1997), and controlling the activity of DnaA (Bramhill and Kornberg, 1988). Dam methyltransferase plays an important role in the first two mechanisms, Figure 7. After the genomic DNA is replicated, it changes from the original fully methylated state to a hemimethylated state. Compared with other 5′GATC3′ sites, the hemimethylation status of eight 5′GATC3′ sequences in the promoter region of *dnaA* and eleven 5′GATC3′ sequences in the *oriC* region of chromosome can be extended to about 10 min (Campbell and Kleckner, 1990). Usually, SeqA bind to the hemimethylated promoter region of *dnaA* before Dam methyltransferase, preventing Dam from acting on those hemimethylated sites and extending their hemimethylated status (Løbner-Olesen et al., 2003). SeqA binding to the hemimethylated 5′GATC3′ of the promoter region of *dnaA* effectively inhibits the transcription of *dnaA* and keep DnaA concentration in the cell at a low level, thereby regulating the initiation of chromosome replication (Campbell and Kleckner, 1990). Further, SeqA also bind to the hemimethylated 5′GATC3′ sequences of *oriC*, preventing DnaA from binding to the origin of replication and preventing the initiation of DNA replication until cell division (Bogan and Helmstetter, 1997). Dam methyltransferase is also necessary for the initiation of chromosome replication in *V. cholerae* (Val et al., 2014). Recent studies have found that *E. coli* Dam methyltransferase can also prevent abnormal *oriC*-independent chromosome replication, also known as constitutive stable DNA replication (cSDR) (Raghunathan et al., 2019).

There are three phases in the progression of the cell cycle in *C. crescentus*: G1, S and G2. CcrM activity has recently been linked to GcrA, a master regulator of the cell cycle that controls the expression of several genes during S-phase (Mohapatra et al., 2014). In *C. crescentus*., three global regulators coordinate the process of the entire cell cycle, DnaA, GcrA and CtrA (Adhikari and Curtis, 2016). DNA methylation by CcrM is involved in the regulatory activity of both GcrA and CtrA, which then affects the regulation of the initiation of chromosome replication initiation and asymmetric division in pre-divisional cells. Reducing the activity of CtrA could make the pre-division length of cells longer (Reisenauer and Shapiro, 2002). On the other hand, phosphorylated CtrA could bind to the origin replication (*Cori*) on the chromosome to prevent the initiation of replication (Mohapatra et al., 2014; Mouammine and Collier, 2018). This also means that CcrM methyltransferase could only indirectly participate in regulating the cell cycle and chromosome replication in *C. crescentus.* (Mouammine and Collier, 2018).

## 6. DNA methylation stabilizes bacterial evolution

DNA methyltransferases play a conservative function to maintain the bacterial genome during bacterial evolution. The R-M systems methylate native DNA and cleave foreign DNA, thereby effectively protecting the DNA of their cell. Studies have suggested that the R-M systems exhibit selfish behaviors and can be used to stabilize plasmids by post-segregational killing (PSK), even when the R-M system encoded by the plasmids persist in their hosts (Naito et al., 1995; Kobayashi, 2001; Oliveira et al., 2014). Further, bacterial R-M systems can distinguish methylation modifications between self DNA and foreign DNA, thereby preventing the horizontal gene transfer (HGT) (Ershova et al., 2015). The flow of genetic information between bacterial cells by HGT drives bacterial evolution, and the R-M systems are key moderators of this process. For example, Dam methyltransferase can also maintain the bacterial genome by inhibiting transposable elements (Tomcsanyi and Berg, 1989) and transposable phage (Murphy et al., 2008).

The R-M systems also maintain DNA mismatch repair (MMR), which is a highly conserved biological pathway that plays a key role in maintaining genomic stability. The specificity of MMR is primarily for base–base mismatches and insertion/deletion mispairs generated during DNA replication and recombination (Li, 2008). During DNA repair, the methyl-directed mismatch repair protein MutH, which belongs to a family of type-II restriction endonucleases, recognizes hemi-methylated DNA sites and removes the nonmethylated daughter DNA strand, ensuring that the methylated parental strand will be used as the template for repair-associated DNA synthesis (Casadesús and Low, 2006).

## 7. Discussion

DNA methyltransferases have been studied for decades now. Partly due to their broad roles and wide varieties, there are still many open questions regarding their function, mechanism and applications.

Due to the nature of DNA methyltransferases (and the R-M systems), it has been difficult to use genetic methods to study whether and how they participate in regulating gene expression. The effects are often global and difficult to pin-point. However, since the discovery and identification of regulators of phase variation, it has been proposed that many R-M systems share similar functions. Moreover, some bacteria contain as many as 50 R-M systems. It is difficult to accept that all 50 R-M systems only function to protect the integrity of bacterial genome (Nobusato et al., 2000). Although many examples of regulators of phase variation involved in regulating gene expression have been found, the mechanisms remain to be elucidated.

The origin and evolution of the R-M systems also raise many questions. Closely related strains have different R-M systems. Sometimes, distantly related species have similar R-M systems, which suggests that the R-M systems undergo horizontal transfer (Oliveira et al., 2014). It is possible that DNA methyltransferases promote bacterial evolution, while also stabilizing it. Studies have observed that bacteria can form subgroups; that is, have the same genotype but different phenotypes (Casadesús and Low, 2013). The formation of subgroups could be used as an adaptation strategy for bacteria to escape their host’s immune system and unfavorable environment, or as a betting-hedging strategy. This risk prevention could improve survival when the environment changes, thus promoting evolution (Beaumont et al., 2009).

The roles of the R-M systems in providing immunity against horizontal gene transfer (HGT) and in stabilizing mobile genetic elements (MGEs) have been much debated. Some studies demonstrate that R-M systems can be inserted into mobile genetic elements (such as plasmids and prophages), and then be horizontally transferred together with the mobile genetic elements (Furuta et al., 2010). However, it is unclear which factors are involved in the process and if there are any triggering factors, internally or externally. It is possible that there is a deep interplay of R-M systems with mobile genetic elements and horizontal transfer (Oliveira et al., 2014).

Although crystal structures of several bacterial DNA methyltransferases have been determined, the target DNA binding region, structure and mechanism have gone unelucidated. In addition, the number of crystal structures obtained is only a tiny portion of the total number of DNA methyltransferases, considering the diversity of these enzymes. High-resolution crystal structures are difficult to obtain, which may reflect certain characteristics of DNA methyltransferases (Kennaway et al., 2009, 2012; Loenen et al., 2014). It is still a mystery that how DNA methyltransferase find their methylation modification sites accurately, given the enormous number of modification sites and the complex 4-dimentional structure present in a genomic DNA. The debate on processive vs. distributive modes of methylation is ongoing.

How does DNA methyltransferase select target bases and what is the driving force for target base inversion? As mentioned before, some DNA methyltransferases are progressive enzymes, and some are partitioning enzymes. It is still unclear how these two functions are differentiated. Is that because of differences in the structure of the enzymes? Understanding the mechanism of DNA methylation may provide opportunities for designing drugs such as methyltransferase inhibitors.

DNA methyltransferases are involved—often critically—in various cellular processes. Because mammals do not methylate DNA at adenine, bacterial MTases that target adenine, such as Dam and CcrM, represent excellent candidates for antibacterial targets. Initial success has been reported with compounds that and selectively targeting bacterial MTases (Mashhoon et al., 2006). It has also been reported that the survival of *E. coli* under the pressure of antibiotics is severely impaired when Dam methylation sites are blocked from modification. Therefore, inhibitors of Dam methyltransferase are likely to improve the efficacy of antibiotics, even if those inhibitors are not antibacterial by themselves (Cohen et al., 2016).

Moreover, DNA methylation modification is associated with various human diseases. Global DNA hypomethylation at CpG islands coupled with local hypermethylation is a hallmark for breast cancer (Shukla et al., 2010; Du et al., 2019). Therefore, methods that can analyze DNA methyltransferase activity with high accuracy and sensitivity may be able to screen for and identify certain diseases. Many new methods for detecting DNA methyltransferase activity have been developed, including electro chemiluminescent, colorimetric, chemiluminescent, fluorescent, and electrochemical (electrochemical) and photoelectrochemical PEC (photoelectrochemical PEC) methods (Yin et al., 2014; Chen et al., 2018; Hou et al., 2019).

Recent improvements in sequencing and other methods have facilitated bacterial epigenomic data collection. Methylome data of more than 2,470 bacteria and archaea have been obtained through single-molecule real-time sequencing and other potential technologies (Fang et al., 2012; Blow et al., 2016; Oliveira et al., 2020). Based on the ubiquity of DNA methyltransferases in bacteria, it is certain that DNA methyltransferases are involved in many more cellular processes than we currently know. People used to think that the epigenetic control of gene expression is the task of orphan methyltransferases. It was not until the emergence of a large number of regulators of phase variation that we realized that this is not the case (Seib et al., 2020). The DNA methyltransferase of a regulator of phase variation can regulate gene expression through epigenetic mechanisms and affect bacterial virulence (Kumar et al., 2018; Estibariz et al., 2019). It is not surprising that the effects of DNA methyltransferases are so far reaching. After a pathogen infects its host, DNA methyltransferases of the pathogen are very likely to modify the host’s genome (Chernov et al., 2015). The phenomenon that pathogens modify the host genome has been reported, but their importance remains to be understood (Niller and Minarovits, 2016; Pereira et al., 2016; Kot et al., 2020).

Bacterial genomes contain numerous DNA methyltransferase modification sites. For example, the genome of *C. crescentus* contains 4,542 CcrM methylation modification sites, but only 23% of these are located inside ORFs. The function and significance of methylation sites outside ORF are not yet fully understood (Kozdon et al., 2013).

It has been proposed that epigenetic memory systems have numerous potential applications in synthetic biology, including life biosensors, death switches or induction systems for industrial protein production. The large variety of bacterial DNA methyltransferases potentially allows for massive multiplexing of signal storage and logical operations depending on more than one input signal. A synthetic epigenetic memory system was designed by using engineered DNA-methylation-sensitive zinc finger proteins to repress a memory operon comprising *ccrM* and a reporter gene (Maier et al., 2017). Development on this frontier will undoubtedly establish new strategies for future bio-chip development.

## Author contributions

QG and SL significantly contributed to the conception and design of the article and interpreting the relevant literature. All authors contributed to the research, drafting and revision of the manuscript and approved the final manuscript.

## Funding

This research was founded by National Natural Science Foundation of China (grant no. 32100029), Natural Science Foundation of Sichuan Province (grant no. 2022NSFSC1677) and Open Project Fund of Mianyang Key Laboratory of Livestock and Poultry Provenance Disease Research (grant no. 202201).

## Conflict of interest

The authors declare that the research was conducted in the absence of any commercial or financial relationships that could be construed as a potential conflict of interest.

## Publisher’s note

All claims expressed in this article are solely those of the authors and do not necessarily represent those of their affiliated organizations, or those of the publisher, the editors and the reviewers. Any product that may be evaluated in this article, or claim that may be made by its manufacturer, is not guaranteed or endorsed by the publisher.

## Footnotes

^1^see http://rebase.neb.com

## References

[ref1] Adamczyk-Poplawska M. Lower M. Piekarowicz A. (2011). Deletion of one nucleotide within the homonucleotide tract present in the hsdS gene alters the DNA sequence specificity of type I restriction-modification system NgoAV. J. Bacteriol. 193, 6750–6759. doi: 10.1128/JB.05672-11, PMID: 21984785 PMC3232900

[ref2] Adhikari S. Curtis P. D. (2016). DNA methyltransferases and epigenetic regulation in bacteria. FEMS Microbiol. Rev. 40, 575–591. doi: 10.1093/femsre/fuw023, PMID: 27476077

[ref3] Ahmad I. Rao D. N. (1996). Functional analysis of conserved motifs in EcoP15I DNA Methyltransferase. J. Mol. Biol. 259, 229–240. doi: 10.1006/jmbi.1996.0315, PMID: 8656425

[ref4] Albert P. Varga B. Zsibrita N. Kiss A. (2018). Circularly permuted variants of two CG-specific prokaryotic DNA methyltransferases. PLoS One 13, e0197232–e0197255. doi: 10.1371/journal.pone.0197232, PMID: 29746549 PMC5944983

[ref5] Albu A. F. Jurkowski T. P. Jeltsch A. (2012). The *Caulobacter crescentus* DNA-(adenine-N6)-methyltransferase CcrM methylates DNA in a distributive manner. Nucleic Acids Res. 40, 1708–1716. doi: 10.1093/nar/gkr768, PMID: 21926159 PMC3287173

[ref6] Ando T. Ishiguro K. Watanabe O. Miyake N. Kato T. Hibi S.. (2010). Restriction-modification systems may be associated with helicobacter pylori virulence. J. Gastroenterol. Hepatol. 25 Suppl 1, S95–S98. doi: 10.1111/j.1440-1746.2009.06211.x, PMID: 20586875

[ref7] Anjum A. Brathwaite K. J. Aidley J. Connerton P. L. Cummings N. J. Parkhill J.. (2016). Phase variation of a Type IIG restriction-modification enzyme alters site-specific methylation patterns and gene expression in campylobacter jejuni strain NCTC11168. Nucleic Acids Res. 44, 4581–4594. doi: 10.1093/nar/gkw019, PMID: 26786317 PMC4889913

[ref8] Anton B. P. Roberts R. J. (2021). Beyond restriction modification: epigenomic roles of DNA methylation in prokaryotes. Annu. Rev. Microbiol. 75, 129–149. doi: 10.1146/annurev-micro-040521-035040, PMID: 34314594

[ref9] Atack J. M. Srikhanta Y. N. Fox K. L. Jurcisek J. A. Brockman K. L. Clark T. A.. (2015). A biphasic epigenetic switch controls immunoevasion, virulence and niche adaptation in non-typeable Haemophilus influenzae. Nat. Commun. 6, 7828–7840. doi: 10.1038/ncomms8828, PMID: 26215614 PMC4525171

[ref10] Atack J. M. Tan A. Bakaletz L. O. Jennings M. P. Seib S. L. (2018). Phasevarions of bacterial pathogens: Methylomics sheds new light on old enemies. Trends Microbiol. 26, 715–726. doi: 10.1016/j.tim.2018.01.008, PMID: 29452952 PMC6054543

[ref11] Attar N. (2016). Bacterial genetics: SMRT-seq reveals an epigenetic switch. Nat. Rev. Microbiol. 14:546. doi: 10.1038/nrmicro.2016.122, PMID: 27477301

[ref12] Baba T. Huan H. Datsenko K. Wanner B. L. Mori H. (2008). The applications of systematic in-frame, single-gene knockout mutant collection of *Escherichia coli* K-12. Methods Mol. Biol. 416, 183–194. doi: 10.1007/978-1-59745-321-9_12, PMID: 18392968

[ref13] Banas J. A. Biswas S. Zhu M. (2011). Effects of DNA methylation on expression of virulence genes in Streptococcus mutans. Appl. Environ. Microbiol. 77, 7236–7242. doi: 10.1128/AEM.00543-11, PMID: 21841035 PMC3194855

[ref14] Bandaru B. Gopal J. Bhagwat A. S. (1996). Overproduction of DNA cytosine methyltransferases causes methylation and C to T mutations at non-canonical sites. J. Biol. Chem. 271, 7851–7859. doi: 10.1074/jbc.271.13.7851, PMID: 8631830

[ref15] Barras F. Marinus M. G. (1989). The great GATC: DNA methylation in *E. coli*. Trends Genet. 5, 139–143. doi: 10.1016/0168-9525(89)90054-1, PMID: 2667217

[ref16] Beaumont H. J. Gallie J. Kost C. Ferguson G. C. Rainey P. B. (2009). Experimental evolution of bet hedging. Nature 462, 90–93. doi: 10.1038/nature08504 19890329

[ref17] Bergerat A. Guschlbauer W. Fazakerley G. V. (1991). Allosteric and catalytic binding of S-adenosylmethionine to *Escherichia coli* DNA adeninemethyltransferasemonitored by 3H NMR. Proc. Natl. Acad. Sci. U. S. A. 88, 6394–6397. doi: 10.1073/pnas.88.15.6394, PMID: 1862071 PMC52091

[ref18] Bheemanaik S. Reddy Y. V. R. Rao D. N. (2006). Structure, function and mechanism of exocyclic DNA methyltransferases. Biochem. J. 399, 177–190. doi: 10.1042/BJ20060854, PMID: 16987108 PMC1609917

[ref19] Bickle T. A. Krüger D. H. (1993). Biology of DNA restriction. Microbiol. Rev. 57, 434–450. doi: 10.1128/mr.57.2.434-450.1993, PMID: 8336674 PMC372918

[ref20] Blakeway L. V. Power P. M. Jen F. E.-C. Worboys S. R. Boitano M. Clark T. A.. (2014). ModM DNA methyltransferase methylome analysis reveals a potential role for Moraxella catarrhalis phasevarions in otitis media. FASEB J. 28, 5197–5207. doi: 10.1096/fj.14-256578, PMID: 25183669

[ref21] Blakeway L. V. Tan A. Lappan R. Ariff A. Pickering J. L. Peacock C. S.. (2018). Moraxella catarrhalis restriction-modification systems are associated with phylogenetic lineage and disease. Genome Biol. Evol. 10, 2932–2946. doi: 10.1093/gbe/evy226, PMID: 30335144 PMC6241649

[ref22] Blow M. J. Clark T. A. Daum C. G. Deutschbauer A. M. Fomenkov A. Fries R.. (2016). The Epigenomic landscape of prokaryotes. PLoS Genet. 12:e1005854. doi: 10.1371/journal.pgen.1005854, PMID: 26870957 PMC4752239

[ref23] Blyn L. B. Braaten B. A. Low D. A. (1990). Regulation of pap pilin phase variation by a mechanism involving differential dam methylation states. EMBO J. 9, 4045–4054. doi: 10.1002/j.1460-2075.1990.tb07626.x, PMID: 2147413 PMC552177

[ref24] Bogan J. A. Helmstetter C. E. (1997). DNA sequestration and transcription in the oriC region of *Escherichia coli*. Mol. Microbiol. 26, 889–896. doi: 10.1046/j.1365-2958.1997.6221989.x, PMID: 9426127

[ref25] Bramhill D. Kornberg A. (1988). A model for initiation at origins of DNA replication. Cells 54, 915–918. doi: 10.1016/0092-8674(88)90102-X, PMID: 2843291

[ref26] Brilli M. Fondi M. Fani R. Mengoni A. Ferri L. Bazzicalupo M.. (2010). The diversity and evolution of cell cycle regulation in alpha-proteobacteria: a comparative genomic analysis. BMC Syst. Biol. 4:52. doi: 10.1186/1752-0509-4-52 20426835 PMC2877005

[ref27] Broadbent S. E. Davies M. R. van der Woude M. W. (2010). Phase variation controls expression of salmonella lipopolysaccharide modification genes by a DNA methylation-dependent mechanism. Mol. Microbiol. 77, 337–353. doi: 10.1111/j.1365-2958.2010.07203.x, PMID: 20487280 PMC2909390

[ref28] Brooks J. E. Blumenthal R. M. Gingeras T. R. (1983). The isolation and characterization of the *Escherichia coli* DNA adenine methylase(dam) gene. Nucleic Acids Res. 11, 837–851. doi: 10.1093/nar/11.3.837, PMID: 6300769 PMC325756

[ref29] Brunet Y. R. Bernard C. S. Gavioli M. Lloubès R. Cascales E. (2011). An epigenetic switch involving overlapping fur and DNA methylation optimizes expression of a type VI secretion gene cluster. PLoS Genet. 7, e1002205–e1002216. doi: 10.1371/journal.pgen.1002205, PMID: 21829382 PMC3145626

[ref30] Callahan S. J. Luyten Y. A. Gupta Y. K. Wilson G. G. Roberts R. J. Morgan R. D.. (2016). Structure of Type IIL restriction-modification enzyme MmeI in complex with DNA has implications for engineering new specificities. PLoS Biol. 14, e1002442–e1002460. doi: 10.1371/journal.pbio.1002442, PMID: 27082731 PMC4833311

[ref31] Campbell J. L. Kleckner N. (1990). *E. coli* oriC and the dnaA gene promoter are sequestered from dam Methyltransfferase following the passage of the chromosomal replication fork. Cells 62, 967–979. doi: 10.1016/0092-8674(90)90271-F, PMID: 1697508

[ref32] Campellone K. G. Roe A. J. Løbner-Olesen A. Murphy K. C. Magoun L. Brady M. J.. (2007). Increased adherence and actin pedestal formation by dam-deficient enterohaemorrhagic *Escherichia coli* O157:H7. Mol. Microbiol. 63, 1468–1481. doi: 10.1111/j.1365-2958.2007.05602.x, PMID: 17302821

[ref33] Casadesús J. (2016). Bacterial DNA methylation and Methylomes. Adv. Exp. Med. Biol. 945, 35–61. doi: 10.1007/978-3-319-43624-1_3 27826834

[ref34] Casadesús J. Low D. (2006). Epigenetic gene regulation in the bacterial world. Microbiol. Mol. Biol. Rev. 70, 830–856. doi: 10.1128/MMBR.00016-06, PMID: 16959970 PMC1594586

[ref35] Casadesús J. Low D. A. (2013). Programmed heterogeneity: epigenetic mechanisms in bacteria. J. Biol. Chem. 288, 13929–13935. doi: 10.1074/jbc.R113.472274, PMID: 23592777 PMC3656251

[ref36] Chen J. Cheng J. Chen X. Inoue M. Liu Y. Song C. (2022). Whole-genome long-read TAPS deciphers DNA methylation patterns at base resolution using PacBio SMRT sequencing technology. Nucleic Acids Res. 50:e104. doi: 10.1093/nar/gkac612, PMID: 35849350 PMC9561279

[ref37] Chen P. Jeannotte R. Weimer B. C. (2014). Exploring bacterial epigenomics in the next-generation sequencing era: a new approach for an emerging frontier. Trends Microbiol. 22, 292–300. doi: 10.1016/j.tim.2014.03.005, PMID: 24725482

[ref38] Chen S. Lv Y. Shen Y. Ji J. Zhou Q. Liu S.. (2018). Highly sensitive and quality self-testable Electrochemiluminescence assay of DNA Methyltransferase activity using multifunctional Sandwich-assembled carbon nitride Nanosheets. ACS Appl. Mater. Interfaces 10, 6887–6894. doi: 10.1021/acsami.7b17813, PMID: 29376630

[ref39] Chen S. Zhang L. Li M. Zhang Y. Sun M. Wang Y.. (2022). Fusobacterium nucleatum reduces METTL3-mediated m(6)a modification and contributes to colorectal cancer metastasis. Nat. Commun. 13:1248. doi: 10.1038/s41467-022-28913-5, PMID: 35273176 PMC8913623

[ref40] Cheng X. Kumar S. Posfai J. Pflugrath J. W. Roberts R. J. (1993). Crystal structure of the Hhal DNA Methyltransferase complexed with S-Adenosyl-L-methionine. Cells 74, 299–307. doi: 10.1016/0092-8674(93)90421-L, PMID: 8343957

[ref41] Cheng X. Roberts R. J. (2001). AdoMet-dependent methylation, DNA methyltransferase and base flipping. Nucleic Acids Res. 29, 3784–3795. doi: 10.1093/nar/29.18.3784, PMID: 11557810 PMC55914

[ref42] Chernov A. V. Reyes L. Xu Z. Gonzalez B. Golovko G. Peterson S.. (2015). Mycoplasma CG- and GATC-specific DNA methyltransferases selectively and efficiently methylate the host genome and alter the epigenetic landscape in human cells. Epigenetics 10, 303–318. doi: 10.1080/15592294.2015.1020000, PMID: 25695131 PMC4623497

[ref43] Clarke J. Wu H. Jayasinghe L. Patel A. Reid S. Bayley H. (2009). Continuous base identification for single-molecule nanopore DNA sequencing. Nat. Nanotechnol. 4, 265–270. doi: 10.1038/nnano.2009.12, PMID: 19350039

[ref44] Cohen N. R. Ross C. A. Jain S. Shapiro R. S. Gutierrez A. Belenky P.. (2016). A role for the bacterial GATC methylome in antibiotic stress survival. Nat. Genet. 48, 581–586. doi: 10.1038/ng.3530, PMID: 26998690 PMC4848143

[ref45] Cota I. Blanc-Potard A. B. Casadesús J. (2012). STM2209-STM2208 (opvAB): a phase variation locus of salmonella enterica involved in control of O-antigen chain length. PLoS One 7, e36863–e36876. doi: 10.1371/journal.pone.0036863, PMID: 22606300 PMC3350482

[ref46] Cota I. Sánchez-Romero M. A. Hernández S. B. Pucciarelli M. G. García-del Portillo F. Casadesús J. (2015). Epigenetic control of salmonella enterica O-antigen chain length: a tradeoff between virulence and bacteriophage resistance. PLoS Genet. 11, e1005667–e1005685. doi: 10.1371/journal.pgen.1005667, PMID: 26583926 PMC4652898

[ref47] Curtis P. D. Brun Y. V. (2014). Identification of essential alphaproteobacterial genes reveals operational variability in conserved developmental and cell cycle systems. Mol. Microbiol. 93, 713–735. doi: 10.1111/mmi.12686, PMID: 24975755 PMC4132054

[ref48] de Ste Croix M. Vacca I. Kwun M. J. Ralph J. D. Bentley S. D. Haigh R.. (2017). Phase-variable methylation and epigenetic regulation by type I restriction-modification systems. FEMS Microbiol. Rev. 41, S3–S15. doi: 10.1093/femsre/fux025, PMID: 28830092

[ref49] Didovyk A. Verdine G. L. (2012). Structural origins of DNA target selection and nucleobase extrusion by a DNA cytosine methyltransferase. J. Biol. Chem. 287, 40099–40105. doi: 10.1074/jbc.M112.413054, PMID: 23012373 PMC3504724

[ref50] Domian I. J. Reisenauer A. Shapiro L. (1999). Feedback control of a master bacterial cell-cycle regulator. Proc. Natl. Acad. Sci. U. S. A. 96, 6648–6653. doi: 10.1073/pnas.96.12.6648, PMID: 10359766 PMC21969

[ref51] Dryden D. T. Murray N. E. Rao D. N. (2001). Nucleoside triphosphate-dependent restriction enzymes. Nucleic Acids Res. 29, 3728–3741. doi: 10.1093/nar/29.18.3728, PMID: 11557806 PMC55918

[ref52] Du Y. Wang S. Li X. Wang Y. Tang A. Kong D. (2019). Terminal deoxynucleotidyl transferase-activated nicking enzyme amplification reaction for specific and sensitive detection of DNA methyltransferase and polynucleotide kinase. Biosens. Bioelectron. 145, 111700–111707. doi: 10.1016/j.bios.2019.111700, PMID: 31539651

[ref53] Dupont C. Armant D. R. Brenner C. A. (2009). Epigenetics: definition, mechanisms and clinical perspective. Semin. Reprod. Med. 27, 351–357. doi: 10.1055/s-0029-1237423, PMID: 19711245 PMC2791696

[ref54] Ershova A. S. Rusinov I. S. Spirin S. A. Karyagina A. S. Alexeevski A. V. (2015). Role of restriction-modification systems in prokaryotic evolution and ecology. Biochemistry (Mosc) 80, 1373–1386. doi: 10.1134/S0006297915100193, PMID: 26567582

[ref55] Estibariz I. Overmann A. Ailloud F. Krebes J. Josenhans C. Suerbaum S. (2019). The core genome m5C methyltransferase JHP1050 (M.Hpy99III) plays an important role in orchestrating gene expression in helicobacter pylori. Nucleic Acids Res. 47, 2336–2348. doi: 10.1093/nar/gky1307, PMID: 30624738 PMC6412003

[ref56] Fälker S. Schilling J. Schmidt M. A. Heusipp G. (2007). Overproduction of DNA adenine methyltransferase alters motility, invasion, and the lipopolysaccharide O-antigen composition of *Yersinia enterocolitica*. Infection Immunnity 75, 4990–4997. doi: 10.1128/IAI.00457-07, PMID: 17682042 PMC2044514

[ref57] Fang G. Munera D. Friedman D. I. Mandlik A. Chao M. C. Banerjee O.. (2012). Genome-wide mapping of methylated adenine residues in pathogenic *Escherichia coli* using single-molecule real-time sequencing. Nat. Biotechnol. 30, 1232–1239. doi: 10.1038/nbt.2432, PMID: 23138224 PMC3879109

[ref58] Fioravanti A. Fumeaux C. Mohapatra S. S. Bompard C. Brilli M. Frandi A.. (2013). DNA binding of the cell cycle transcriptional regulator GcrA depends on N6-adenosine methylation in Caulobacter crescentus and other Alphaproteobacteria. PLoS Genet. 9:e1003541. doi: 10.1371/journal.pgen.1003541, PMID: 23737758 PMC3667746

[ref59] Flusberg B. A. Webster D. R. Lee J. H. Travers K. J. Olivares E. C. Clark T. A.. (2010). Direct detection of DNA methylation during single-molecule, real-time sequencing. Nat. Methods 7, 461–465. doi: 10.1038/nmeth.1459, PMID: 20453866 PMC2879396

[ref60] Fox K. L. Dowideit S. J. Erwin A. L. Srikhanta Y. N. Smith A. L. Jennings M. P. (2007). Haemophilus influenzae phasevarions have evolved from type III DNA restriction systems into epigenetic regulators of gene expression. Nucleic Acids Res. 35, 5242–5252. doi: 10.1093/nar/gkm571, PMID: 17675301 PMC1976455

[ref61] Furuta Y. Abe K. Kobayashi I. (2010). Genome comparison and context analysis reveals putative mobile forms of restriction-modification systems and related rearrangements. Nucleic Acids Res. 38, 2428–2443. doi: 10.1093/nar/gkp1226, PMID: 20071371 PMC2853133

[ref62] García-Pastor L. Sánchez-Romero M. A. Gutiérrez G. Puerta-Fernández E. Casadesús J. (2018). Formation of phenotypic lineages in salmonella enterica by a pleiotropic fimbrial switch. PLoS Genet. 14:e1007677. doi: 10.1371/journal.pgen.1007677, PMID: 30252837 PMC6173445

[ref63] García-Pastor L. Sánchez-Romero M. A. Jakomin M. Puerta-Fernández E. Casadesús J. (2019). Regulation of bistability in the std fimbrial operon of salmonella enterica by DNA adenine methylation and transcription factors HdfR, StdE and StdF. Nucleic Acids Res. 47, 7929–7941. doi: 10.1093/nar/gkz530, PMID: 31216025 PMC6735912

[ref64] Gauntlett J. C. Nilsson H. Fulurija A. Marshall B. J. Benghezal M. (2014). Phase-variable restriction/modification systems are required for helicobacter pylori colonization. Gut. Pathogeens 6, 35–40. doi: 10.1186/s13099-014-0035-z, PMID: 25349630 PMC4209511

[ref65] Giacomodonato M. N. Sarnacki S. H. Llana M. N. Cerquetti M. C. (2009). Dam and its role in pathogenicity of salmonella enterica. J. Infect. Dev. Ctries. 3, 484–490. doi: 10.3855/jidc.465, PMID: 19762965

[ref66] Goedecke K. Pignot M. Goody R. S. Scheidig A. J. Weinhold E. (2001). Structure of the N6-adenine DNA methyltransferase M•TaqI in complex with DNA and a cofactor analog. Nat. Struct. Biol. 8, 121–125. doi: 10.1038/84104, PMID: 11175899

[ref67] Gong W. O’Gara M. Blumenthal R. M. Cheng X. (1997). Structure of PvuII DNA-(cytosine N4) methyltransferase, an example of domain permutation and protein fold assignment. Nucleic Acids Res. 25, 2702–2715. doi: 10.1093/nar/25.14.2702, PMID: 9207015 PMC146797

[ref68] Gonzalez D. Collier J. (2013). DNA methylation by CcrM activates the transcription of two genes required for the division of *Caulobacter crescentus*. Mol. Microbiol. 88, 203–218. doi: 10.1111/mmi.12180, PMID: 23480529 PMC3708114

[ref69] Gonzalez D. Kozdon J. B. McAdams H. H. Shapiro L. Collier J. (2014). The functions of DNA methylation by CcrM in *Caulobacter crescentus*: a global approach. Nucleic Acids Res. 42, 3720–3735. doi: 10.1093/nar/gkt1352 24398711 PMC3973325

[ref70] Green L. R. Dave N. Adewoye A. B. Lucidarme J. Clark S. A. Oldfield N. J.. (2019). Potentiation of phase variation in multiple outer-membrane proteins during spread of the Hyperinvasive Neisseria meningitidis serogroup W ST-11 lineage. J. Infect. Dis. 220, 1109–1117. doi: 10.1093/infdis/jiz275, PMID: 31119276 PMC6735796

[ref71] Gupta Y. K. Chan S. Xu S. Aggarwal A. K. (2015). Structural basis of asymmetric DNA methylation and ATP-triggered long-range diffusion by EcoP15I. Nat. Commun. 6, 7363–7373. doi: 10.1038/ncomms8363, PMID: 26067164 PMC4490356

[ref72] Han S. Liu J. Li M. Zhang Y. Duan X. Zhang Y.. (2022). DNA Methyltransferase regulates nitric oxide homeostasis and virulence in a chronically adapted Pseudomonas aeruginosa strain. mSystems 7:e0043422. doi: 10.1128/msystems.00434-22, PMID: 36106744 PMC9600465

[ref73] Heithoff D. M. House J. K. Thomson P. C. Mahan M. J. (2015). Development of a salmonella cross-protective vaccine for food animal production systems. Vaccine 33, 100–107. doi: 10.1016/j.vaccine.2014.11.012, PMID: 25448106

[ref74] Hénaut A. Rouxel T. Gleizes A. Moszer I. Danchin A. (1996). Uneven distribution of GATC motifs in the *Escherichia coli* chromosome, its plasmids and its phages. J. Mol. Biol. 257, 574–585. doi: 10.1006/jmbi.1996.0186, PMID: 8648625

[ref75] Hernday A. D. Braaten B. A. Broitman-Maduro G. Engelberts P. Low D. A. (2004). Regulation of the pap epigenetic switch by CpxAR: phosphorylated CpxR inhibits transition to the phase ON state by competition with Lrp. Mol. Cell 16, 537–547. doi: 10.1016/j.molcel.2004.10.020, PMID: 15546614

[ref76] Holtzendorff J. Hung D. Brende P. Reisenauer A. Viollier P. H. McAdams H. H.. (2004). Oscillating global regulators control the genetic circuit driving a bacterial cell cycle. Science 304, 983–987. doi: 10.1126/science.1095191, PMID: 15087506

[ref77] Hong S. Cheng X. (2016). DNA Base flipping: a general mechanism for writing, Reading, and erasing DNA modifications. Adv. Exp. Med. Biol. 945, 321–341. doi: 10.1007/978-3-319-43624-1_14, PMID: 27826845 PMC5542066

[ref78] Horton J. R. Liebert K. Bekes M. Jeltsch A. Cheng X. (2006). Structure and substrate recognition of the *Escherichia coli* DNA adenine methyltransferase. J. Mol. Biol. 358, 559–570. doi: 10.1016/j.jmb.2006.02.028, PMID: 16524590 PMC2672621

[ref79] Horton J. R. Woodcockm C. B. Opot S. B. Reich N. O. Zhang X. Cheng X. (2019). The cell cycle-regulated DNA adenine methyltransferase CcrM opens a bubble at its DNA recognition site. Nat. Commun. 10, 4600–4609. doi: 10.1038/s41467-019-12498-7 31601797 PMC6787082

[ref80] Horton J. R. Zhang X. Blumenthal R. M. Cheng X. (2015). Structures of *Escherichia coli* DNA adenine methyltransferase (dam) in complex with a non-GATC sequence: potential implications for methylation-independent transcriptional repression. Nucleic Acids Res. 43, 4296–4308. doi: 10.1093/nar/gkv251, PMID: 25845600 PMC4417163

[ref81] Hou T. Xu N. Wang W. Ge L. Li F. (2019). Label-free and immobilization-free photoelectrochemical biosensing strategy using methylene blue in homogeneous solution as signal probe for facile DNA methyltransferase activity assay. Biosens. Bioelectron. 141, 111395–111401. doi: 10.1016/j.bios.2019.111395, PMID: 31195203

[ref82] Kahng L. S. Shapiro L. (2001). The CcrM DNA methyltransferase of agrobacterium tumefaciens is essential, and its activity is cell cycle regulated. J. Bacteriol. 183, 3065–3075. doi: 10.1128/JB.183.10.3065-3075.2001, PMID: 11325934 PMC95206

[ref83] Kahramanoglou C. Prieto A. I. Khedkar S. Haase B. Gupta A. Benes V.. (2012). Genomics of DNA cytosine methylation in *Escherichia coli* reveals its role in stationary phase transcription. Nat. Commun. 3, 886–895. doi: 10.1038/ncomms1878, PMID: 22673913

[ref84] Kennaway C. K. Obarska-Kosinska A. White J. H. Tuszynska I. Cooper L. P. Bujnicki J. M.. (2009). The structure of M.EcoKI Type I DNA methyltransferase with a DNA mimic antirestriction protein. Nucleic Acids Res. 37, 762–770. doi: 10.1093/nar/gkn988, PMID: 19074193 PMC2647291

[ref85] Kennaway C. K. Taylor J. E. Song C. F. Potrzebowski W. Nicholson W. White J. H.. (2012). Structure and operation of the DNA-translocating type I DNA restriction enzymes. Genes Dev. 26, 92–104. doi: 10.1101/gad.179085.111, PMID: 22215814 PMC3258970

[ref86] Kobayashi I. (2001). Behavior of restriction-modification systems as selfish moblie elements and theri impact on genome evolution. Nucleic Acids Res. 29, 3742–3756. doi: 10.1093/nar/29.18.3742, PMID: 11557807 PMC55917

[ref87] Kot W. Olsen N. S. Nielsen T. K. Hutinet G. de Crécy-Lagard V. Cui L.. (2020). Detection of preQ0 deazaguanine modifications in bacteriophage CAjan DNA using Nanopore sequencing reveals same hypermodification at two distinct DNA motifs. Nucleic Acids Res. 48, 10383–10396. doi: 10.1093/nar/gkaa735, PMID: 32941607 PMC7544227

[ref88] Kozdon J. B. Melfi M. D. Luong K. Clark T. A. Boitano M. Wang S.. (2013). Global methylation state at base-pair resolution of the Caulobacter genome throughout the cell cycle. Proc. Natl. Acad. Sci. U. S. A. 110, E4658–E4667. doi: 10.1073/pnas.1319315110, PMID: 24218615 PMC3845142

[ref89] Kulkarni M. Niewan N. van Aelst K. Szczelkun M. D. Saikrishnan K. (2016). Structural insights into DNA sequence recognition by Type ISP restriction-modification enzymes. Nucleic Acids Res. 44, 4396–4408. doi: 10.1093/nar/gkw154, PMID: 26975655 PMC4872093

[ref90] Kumar S. Karmakar B. C. Nagarajan D. Mukhopadhyay A. K. Morgan R. D. Rao D. N. (2018). N4-cytosine DNA methylation regulates transcription and pathogenesis in helicobacter pylori. Nucleic Acids Res. 46, 3429–3445. doi: 10.1093/nar/gky126, PMID: 29481677 PMC5909468

[ref91] Kwiatek A. Mrozek A. Bacal P. Piekarowicz A. Adamczyk-Popławska M. Type I. I. I.. (2015). NgoAX from Neisseria gonorrhoeae FA1090 regulates biofilm formation and interactions with human cells. Front. Microbiol. 6, 1426–1442. doi: 10.3389/fmicb.2015.01426, PMID: 26733970 PMC4685087

[ref92] Laub M. T. Chen S. L. Shapiro L. McAdams H. H. (2002). Genes directly controlled by CtrA, a master regulator of the Caulobacter cell cycle. Proc. Natl. Acad. Sci. U. S. A. 99, 4632–4637. doi: 10.1073/pnas.062065699, PMID: 11930012 PMC123699

[ref93] Lauster R. Trautner T. A. Noyer-Weidner M. (1989). Cytosine-specific Type II DNA methyltransferases a conserved enzyme Core with variable target-recognizing domains. J. Mol. Biol. 206, 305–312. doi: 10.1016/0022-2836(89)90480-4, PMID: 2716049

[ref94] Lees J. Gladstone R. A. (2015). R-M systems go on the offensive. Nat. Rev. Microbiol. 13:131. doi: 10.1038/nrmicro3435, PMID: 25639682

[ref95] Li G. M. (2008). Mechanisms and functions of DNA mismatch repair. Cell Res. 18, 85–98. doi: 10.1038/cr.2007.115, PMID: 18157157

[ref96] Li J. Zhang J. (2019). Phase variation of Streptococcus pneumoniae. Microbiol. Spectrum 7, 1–16. doi: 10.1128/microbiolspec.GPP3-0005-2018 PMC11590436 30737916

[ref97] Liebert K. Horton J. R. Chahar S. Orwick M. Cheng X. Jeltsch A. (2007). Two alternative conformations of S-adenosyl-L-homocysteine bound to *Escherichia coli* DNA adenine methyltransferase and the implication of conformational changes in regulating the catalytic cycle. J. Biol. Chem. 282, 22848–22855. doi: 10.1074/jbc.M700926200, PMID: 17545164

[ref98] Lin L. F. Posfai J. Roberts R. J. Kong H. (2001). Comparative genomics of the restriction-modification systems in helicobacter pylori. Proc. Natl. Acad. Sci. U. S. A. 98, 2740–2745. doi: 10.1073/pnas.051612298, PMID: 11226310 PMC30209

[ref99] Liu Y. Tang Q. Zhang J. Tian L. Gao P. Yan X. (2017). Structural basis underlying complex assembly and conformational transition of the type I R-M system. Proc. Natl. Acad. Sci. U. S. A. 114, 11151–11156. doi: 10.1073/pnas.1711754114, PMID: 28973912 PMC5651777

[ref100] Løbner-Olesen A. Boye E. Marinus M. G. (1992). Expression of the *Escherichia coli* dam gene. Mol. Microbiol. 6, 1841–1851. doi: 10.1111/j.1365-2958.1992.tb01356.x, PMID: 1630320

[ref101] Løbner-Olesen A. Marinus M. G. Hansen F. G. (2003). Role of SeqA and dam in *Escherichia coli* gene expression: a global/microarray analysis. Proc. Natl. Acad. Sci. U. S. A. 100, 4672–4677. doi: 10.1073/pnas.0538053100, PMID: 12682301 PMC153614

[ref102] Løbner-Olesen A. Skovgarrd O. Marinus M. G. (2005). Dam methylation: coordinating cellular processes. Curr. Opin. Microbiol. 8, 154–160. doi: 10.1016/j.mib.2005.02.009, PMID: 15802246

[ref103] Loenen W. A. M. Dryden D. T. F. Raleigh E. A. Wilson G. G. (2014). Type I restriction enzymes and their relatives. Nucleic Acids Res. 42, 20–44. doi: 10.1093/nar/gkt847, PMID: 24068554 PMC3874165

[ref104] López-Garrido J. Casadesús J. (2010). Regulation of salmonella enterica pathogenicity island 1 by DNA adenine methylation. Genetics 184, 637–649. doi: 10.1534/genetics.109.108985, PMID: 20008574 PMC2845334

[ref105] Lu M. Campbell J. L. Boye E. Kleckner N. (1994). SeqA: a negative modulator of replication initiation in *E. coli*. Cells 77, 413–426. doi: 10.1016/0092-8674(94)90156-2, PMID: 8011018

[ref106] Lüneberg E. Zähringer U. Knirel Y. A. Steinmann D. Hartmann M. Steinmetz I.. (1998). Phase-variable expression of lipopolysaccharide contributes to the virulence of legionella pneumophila. J. Exp. Med. 188, 49–60. doi: 10.1084/jem.188.1.49, PMID: 9653083 PMC2525541

[ref107] Lyko F. (2018). The DNA methyltransferase family: a versatile toolkit for epigenetic regulation. Nat. Rev. Genetics 19, 81–92. doi: 10.1038/nrg.2017.80, PMID: 29033456

[ref108] Ma B. Ma J. Liu D. Guo L. Chen H. Ding J.. (2016). Biochemical and structural characterization of a DNA N6-adenine methyltransferase from helicobacter pylori. Oncotarget 7, 40965–40977. doi: 10.18632/oncotarget.9692, PMID: 27259995 PMC5173035

[ref109] Maier J. A. H. Möhrle R. Jeltsch A. (2017). Design of synthetic epigenetic circuits featuring memory effects and reversible switching based on DNA methylation. Nat. Commun. 8, 15336–15346. doi: 10.1038/ncomms15336, PMID: 28537256 PMC5458116

[ref110] Manso A. S. Chai M. H. Atack J. M. Furi L. de Ste Croix M. Haigh R.. (2014). A random six-phase switch regulates pneumococcal virulence via global epigenetic changes. Nat. Commun. 5, 5055–5065. doi: 10.1038/ncomms6055, PMID: 25268848 PMC4190663

[ref111] Marinus M. G. Casadesús J. (2009). Roles of DNA adenine methylation in host-pathogen interactions: mismatch repair, transcriptional regulation, and more. FEMS Microbiol. Rev. 33, 488–503. doi: 10.1111/j.1574-6976.2008.00159.x, PMID: 19175412 PMC2941194

[ref112] Marinus M. G. Morris N. R. (1973). Isolation of deoxyribonucleic acid Methylase mutants of *Escherichia coli* K-12. J. Bacteriol. 114, 1143–1150. doi: 10.1128/jb.114.3.1143-1150.1973, PMID: 4576399 PMC285375

[ref113] Mashhoon N. Pruss C. Carroll M. Johnson P. H. Reich N. O. (2006). Selective inhibitors of bacterial DNA adenine methyltransferases. J. Biomol. Screen. 11, 497–510. doi: 10.1177/1087057106287933, PMID: 16760373

[ref114] Matthews M. M. Thomas J. M. Zheng Y. Tran K. Phelps K. J. Scott A. I.. (2016). Structures of human ADAR2 bound to dsRNA reveal base-flipping mechanism and basis for site selectivity. Nat. Struct. Mol. Biol. 23, 426–433. doi: 10.1038/nsmb.3203, PMID: 27065196 PMC4918759

[ref115] Militello K. T. Finnerty-Haggerty L. Kambhampati O. Huss R. Knapp R. (2020). DNA cytosine methyltransferase enhances viability during prolonged stationary phase in *Escherichia coli*. FEMS Microbiol. Lett. 367:fnaa166. doi: 10.1093/femsle/fnaa166, PMID: 33045036

[ref116] Militello K. T. Mandarano A. H. Varechtchouk O. Simon R. D. (2014). Cytosine DNA methylation influences drug resistance in *Escherichia coli* through increased sugE expression. FEMS Microbiol Letter 350, 100–106. doi: 10.1111/1574-6968.12299, PMID: 24164619

[ref117] Mohapatra S. S. Fioravanti A. Biondi E. G. (2014). DNA methylation in Caulobacter and other Alphaproteobacteria during cell cycle progression. Trends Microbiology 22, 528–535. doi: 10.1016/j.tim.2014.05.003, PMID: 24894626

[ref118] Morita R. Ishikawa H. Nakagawa N. Kuramitsu S. Masui R. (2008). Crystal structure of a putative DNA methylase TTHA0409 from Thermus thermophilus HB8. Proteins 73, 259–264. doi: 10.1002/prot.22158, PMID: 18618709

[ref119] Mouammine A. Collier J. (2018). The impact of DNA methylation in Alphaproteobacteria. Molecular Microbiology l 110, 1–10. doi: 10.1111/mmi.14079, PMID: 29995343

[ref120] Murphy J. Mahony J. Ainsworth S. Nauta A. van Sinderen D. (2013). Bacteriophage orphan DNA methyltransferases: insights from their bacterial origin, function, and occurrence. Appl. Environ. Microbiol. 79, 7547–7555. doi: 10.1128/AEM.02229-13, PMID: 24123737 PMC3837797

[ref121] Murphy K. C. Ritchie J. M. Waldor M. K. Løbner-Olesen A. Marinus M. G. (2008). Dam methyltransferase is required for stable lysogeny of the Shiga toxin (Stx2)-encoding bacteriophage 933W of enterohemorrhagic *Escherichia coli* O157:H7. J. Bacteriol. 190, 438–441. doi: 10.1128/JB.01373-07, PMID: 17981979 PMC2223730

[ref122] Murray N. E. (2000). Type I restriction systems: sophisticated molecular machines (a legacy of Bertani and Weigle). Microbiol. Mol. Biol. Rev. 64, 412–434. doi: 10.1128/MMBR.64.2.412-434.2000, PMID: 10839821 PMC98998

[ref123] Murray I. A. Morgan R. D. Luyten Y. Fomenkov A. Correa I. R. Dai N.. (2018). The non-specific adenine DNA methyltransferase M.EcoGII. Nucleic Acids Res. 46, 840–848. doi: 10.1093/nar/gkx1191, PMID: 29228259 PMC5778455

[ref124] Naito T. Kusano K. Kobayashi I. (1995). Selfish behavior of restriction-modification systems. Science 267, 897–899. doi: 10.1126/science.7846533, PMID: 7846533

[ref125] Narayanan N. Banerjee A. Jain D. Kulkarni D. S. Sharma R. Nirwal S.. (2020). Tetramerization at Low pH licenses DNA methylation activity of M.HpyAXI in the presence of acid stress. J. Mol. Biol. 432, 324–342. doi: 10.1016/j.jmb.2019.10.001, PMID: 31628946

[ref126] Niller H. H. Minarovits J. (2016). Patho-epigenetics of infectious diseases caused by intracellular bacteria. Adv. Exp. Med. Biol. 879, 107–130. doi: 10.1007/978-3-319-24738-0_6, PMID: 26659266

[ref127] Nobusato A. Uchiyama I. Kobayashi I. (2000). Diversity of restriction–modification gene homologues in helicobacter pylori. Gene 259, 89–98. doi: 10.1016/S0378-1119(00)00455-8, PMID: 11163966

[ref128] Nou X. Braaten B. Kaltenbach L. Low D. A. (1995). Differential binding of Lrp to two sets of pap DNA binding sites mediated by Papi regulates pap phase variationin *Escherichia coli*. EMBO J. 14, 5785–5797. doi: 10.1002/j.1460-2075.1995.tb00267.x, PMID: 8846772 PMC394697

[ref129] O'Gara M. Zhang X. Roberts R. J. Cheng X. (1999). Structure of a binary complex of HhaI Methyltransferase with S-adenosyl-L-methionine formed in the presence of a short non-specific DNA oligonucleotide. J. Mol. Biol. 287, 201–209. doi: 10.1006/jmbi.1999.2608, PMID: 10080885

[ref130] Oliveira P. H. Ribis J. W. Garrett E. M. Trzilova D. Kim A. Sekulovic O.. (2020). Epigenomic characterization of Clostridioides difficile finds a conserved DNA methyltransferase that mediates sporulation and pathogenesis. Nat. Microbiol. 5, 166–180. doi: 10.1038/s41564-019-0613-4, PMID: 31768029 PMC6925328

[ref131] Oliveira P. H. Touchon M. Rocha E. P. C. (2014). The interplay of restriction-modification systems with mobile genetic elements and their prokaryotic hosts. Nucleic Acids Res. 42, 10618–10631. doi: 10.1093/nar/gku734, PMID: 25120263 PMC4176335

[ref132] Oshima T. Wada C. Kawagoe Y. Ara T. Maeda M. Masuda Y.. (2002). Genome-wide analysis of deoxyadenosine methyltransferase-mediated control of gene expression in *Escherichia coli*. Mol. Microbiol. 45, 673–695. doi: 10.1046/j.1365-2958.2002.03037.x, PMID: 12139615

[ref133] Osipiuk J. Walsh M. A. Joachimiak A. (2003). Crystal structure of MboIIA methyltransferase. Nucleic Acids Res. 31, 5440–5448. doi: 10.1093/nar/gkg713, PMID: 12954781 PMC203307

[ref134] Owen P. Meehan M. de Loughry-Doherty H. Henderson I. (1996). Phase-variable outer membrane proteins in *Escherichia coli*. FEMS Immunol. Med. Microbiol. 16, 63–76. doi: 10.1111/j.1574-695X.1996.tb00124.x, PMID: 8988388

[ref135] Park S. Lee H. Song J. Sun J. Hwang H. Nishi K.. (2012). Structural characterization of a modification subunit of a putative type I restriction enzyme from Vibrio vulnificus YJ016. Acta Crystallogr D Biol. Crystallogr. 68, 1570–1577. doi: 10.1107/S0907444912038826, PMID: 23090406

[ref136] Pereira J. M. Hamon M. A. Cossart P. (2016). A lasting impression: epigenetic memory of bacterial infections? Cell Host Microbe 19, 579–582. doi: 10.1016/j.chom.2016.04.012, PMID: 27173925

[ref137] Pósfai J. Bhagwat A. S. Pósfai G. Roberts R. J. (1989). Predictive motifs derived from cytosine methyltransferases. Nucleic Acids Res. 17, 2421–2435. doi: 10.1093/nar/17.7.2421, PMID: 2717398 PMC317633

[ref138] Pucciarelli M. G. Prieto A. I. Casadesús J. García-del Portillo F. (2002). Envelope instability in DNA adenine methylase mutants of salmonella enterica. Microbiology 148, 1171–1182. doi: 10.1099/00221287-148-4-1171, PMID: 11932461

[ref139] Raghunathan N. Goswami S. Leela J. K. Pandiyan A. Gowrishankar J. (2019). A new role for *Escherichia coli* dam DNA methylase in prevention of aberrant chromosomal replication. Nucleic Acids Res. 47, 5698–5711. doi: 10.1093/nar/gkz242, PMID: 30957852 PMC6582345

[ref140] Reich N. O. Dang E. Kurnik M. Pathuri S. Woodcock C. B. (2018). The highly specific, cell cycle-regulated methyltransferase from *Caulobacter crescentus* relies on a novel DNA recognition mechanism. J. Biol. Chem. 293, 19038–19046. doi: 10.1074/jbc.RA118.005212, PMID: 30323065 PMC6295719

[ref141] Reinisch K. M. Chen L. Verdine G. L. Lipscomb W. N. (1995). The crystal structure of Haelll Methyltransferase covalently complexed to DNA: an Extrahelical cytosine and Rearranged Base pairing. Cells 82, 143–153. doi: 10.1016/0092-8674(95)90060-8, PMID: 7606780

[ref142] Reisenauer A. Quon K. Shapiro L. (1999). The CtrA response regulator mediates temporal control of gene expression during the Caulobacter cell cycle. J. Bacteriol. 181, 2430–2439. doi: 10.1128/JB.181.8.2430-2439.1999, PMID: 10198005 PMC93667

[ref143] Reisenauer A. Shapiro L. (2002). DNA methylation affects the cell cycle transcription of the CtrA global regulator in Caulobacter. EMBO J. 21, 4969–4977. doi: 10.1093/emboj/cdf490, PMID: 12234936 PMC126286

[ref144] Roberts R. J. Cheng X. (1998). Base Flipping. Annu. Rev. Biochem. 67, 181–198. doi: 10.1146/annurev.biochem.67.1.181, PMID: 9759487

[ref145] Roberts D. Hoopes B. C. McClure W. R. Kleckner N. (1985). IS10 transposition IS regulated by DNA adenine methylation. Cells 43, 117–130. doi: 10.1016/0092-8674(85)90017-0, PMID: 3000598

[ref146] Roberts R. J. Vincze T. Posfai J. Macelis D. (2015). REBASE–a database for DNA restriction and modification: enzymes, genes and genomes. Nucleic Acids Res. 43, D298–D299. doi: 10.1093/nar/gku1046, PMID: 25378308 PMC4383893

[ref147] Robertson G. T. Reisenauer A. Wright R. Jensen R. B. Jensen A. Shapiro L.. (2000). The Brucella abortus CcrM DNA Methyltransferase is essential for viability, and its overexpression attenuates intracellular replication in murine macrophages. J. Bacteriol. 182, 3482–3489. doi: 10.1128/JB.182.12.3482-3489.2000, PMID: 10852881 PMC101938

[ref148] Robinson V. L. Oyston P. C. F. Titball R. W. (2005). A dam mutant of Yersinia pestis is attenuated and induces protection against plague. FEMS Microbiol. Lett. 252, 251–256. doi: 10.1016/j.femsle.2005.09.001, PMID: 16188402

[ref149] Roth M. Helm-Kruse S. Friedrich T. Jeltsch A. (1998). Functional roles of conserved amino acid residues in DNA methyltransferases investigated by site-directed mutagenesis of the EcoRV adenine-N6-methyltransferase. J. Biol. Chem. 273, 17333–17342. doi: 10.1074/jbc.273.28.17333, PMID: 9651316

[ref150] Sánchez-Romero M. A. Casadesús J. (2020). The bacterial epigenome. Nat. Rev. Microbiol. 18, 7–20. doi: 10.1038/s41579-019-0286-2, PMID: 31728064

[ref151] Sánchez-Romero M. A. Cota I. Casadesús J. (2015). DNA methylation in bacteria: from the methyl group to the methylome. Curr. Opin. Microbiol. 25, 9–16. doi: 10.1016/j.mib.2015.03.004, PMID: 25818841

[ref152] Sankpal U. T. Rao D. N. (2002). Structure, function, and mechanism of HhaI DNA methyltransferases. Crit. Rev. Biochem. Mol. Biol. 37, 167–197. doi: 10.1080/10409230290771492 12139442

[ref153] Scavetta R. D. Thomas C. B. Walsh M. A. Szegedi S. Joachimiak A. Gumport R. I.. (2000). Structure of Rsrl methyltransferase, a member of the N6-adenine β class of DNA methyltramsferases. Nucleic Acids Res. 28, 3950–3961. doi: 10.1093/nar/28.20.3950, PMID: 11024175 PMC110776

[ref154] Schluckebier G. Labahn J. Granzin J. Schildkraut I. Saenger W. (1995). A model for DNA binding and enzyme action derived from crystallographic studies of the TaqIN6-adenine-methyltransferase. Gene 157, 131–134. doi: 10.1016/0378-1119(94)00690-T, PMID: 7607476

[ref155] Seib K. L. Pigozzi E. Muzzi A. Gawthorne J. A. Delany I. Jennings M. P.. (2011). A novel epigenetic regulator associated with the hypervirulent Neisseria meningitidis clonal complex 41/44. FASEB J. 25, 3622–3633. doi: 10.1096/fj.11-183590, PMID: 21680891

[ref156] Seib K. L. Srikhanta Y. N. Atack J. M. Jennings M. P. (2020). Epigenetic regulation of virulence and Immunoevasion by phase-variable restriction-modification Systems in Bacterial Pathogens. Annu. Rev. Microbiol. 74, 655–671. doi: 10.1146/annurev-micro-090817-062346, PMID: 32689914

[ref157] Seshasayee A. S. N. (2007). An assessment of the role of DNA adenine methyltransferase on gene expression regulation in *E coli*. PLoS One 2, e273–e277. doi: 10.1371/journal.pone.0000273, PMID: 17342207 PMC1804101

[ref158] Seshasayee A. S. N. Singh P. Krishna S. (2012). Context-dependent conservation of DNA methyltransferases in bacteria. Nucleic Acids Res. 40, 7066–7073. doi: 10.1093/nar/gks390, PMID: 22573173 PMC3424554

[ref159] Shen B. W. Xu D. Chan S. Zheng Y. Zhu Z. Xu S.. (2011). Characterization and crystal structure of the type IIG restriction endonuclease RM.BpuSI. Nucleic Acids Res. 39, 8223–8236. doi: 10.1093/nar/gkr543, PMID: 21724614 PMC3185434

[ref160] Shier V. K. Hancery C. J. Benkovic S. J. (2001). Identification of the active oligomeric state of an essential adenine DNA methyltransferase from *Caulobacter crescentus*. J. Biol. Chem. 276, 14744–14751. doi: 10.1074/jbc.M010688200, PMID: 11278726

[ref161] Shukla V. Coumoul X. Lahusen T. Wang R. Xu X. Vassilopoulos A.. (2010). BRCA1 affects global DNA methylation through regulation of DNMT1. Cell Res. 20, 1201–1215. doi: 10.1038/cr.2010.128, PMID: 20820192 PMC9423198

[ref162] Skerker J. M. Laub M. T. (2004). Cell-cycle progression and the generation of asymmetry in *Caulobacter crescentus*. Nat. Rev. Microbiol. 2, 325–337. doi: 10.1038/nrmicro864, PMID: 15031731

[ref163] Smith H. O. Annau T. M. Chandrasegaran S. (1990). Finding sequence motifs in groups of functionally related proteins. Proc. Natl. Acad. Sci. U. S. A. 87, 826–830. doi: 10.1073/pnas.87.2.826, PMID: 1689055 PMC53359

[ref164] Sobetzko P. Jelonek L. Strickert M. Han W. Goesmann A. Waldminghaus T. (2016). DistAMo: a web-based tool to characterize DNA-motif distribution on bacterial chromosomes. Front. Microbiol. 7, 283–294. doi: 10.3389/fmicb.2016.00283, PMID: 27014208 PMC4786541

[ref165] Srikhanta Y. N. Dowideit S. J. Edwards J. L. Falsetta M. L. Wu H. J. Harrison O. B.. (2009). Phasevarions mediate random switching of gene expression in pathogenic Neisseria. PLoS Pathog. 5, e1000400–e1000422. doi: 10.1371/journal.ppat.1000400, PMID: 19390608 PMC2667262

[ref166] Srikhanta Y. N. Fox K. L. Jennings M. P. (2010). The phasevarion: phase variation of type III DNA methyltransferases controls coordinated switching in multiple genes. Nat. Rev. Microbiol. 8, 196–206. doi: 10.1038/nrmicro2283, PMID: 20140025

[ref167] Srikhanta Y. N. Gorrell R. J. Power P. M. Tsyganov K. Boitano M. Clark T. A.. (2017). Methylomic and phenotypic analysis of the ModH5 phasevarion of helicobacter pylori. Sci. Rep. 7, 16140–16154. doi: 10.1038/s41598-017-15721-x, PMID: 29170397 PMC5700931

[ref168] Srikhanta Y. N. Maguire T. L. Stacey K. J. Grimmond S. M. Jennings M. P. (2005). The phasevarion: a genetic system controlling coordinated, random switching of expression of multiple genes. Proc. Natl. Acad. Sci. U. S. A. 102, 5547–5551. doi: 10.1073/pnas.0501169102, PMID: 15802471 PMC556257

[ref169] Stephens C. Reisenauer A. Wright R. Shapiro L. (1996). A cellcycle-regulatedbacterialDNA methyltransferaseis essential for viability. Proc. Natl. Acad. Sci. U. S. A. 93, 1210–1214. doi: 10.1073/pnas.93.3.1210, PMID: 8577742 PMC40058

[ref170] Tan A. Atack J. M. Jennings M. P. Seib K. L. (2016). The capricious nature of bacterial pathogens: Phasevarions and vaccine development. Front. Immunol. 7:586. doi: 10.3389/fimmu.2016.00586, PMID: 28018352 PMC5149525

[ref171] Tauseef I. Harrison O. B. Wooldridge K. G. Feavers I. M. Neal K. R. Gray S. J.. (2011). Influence of the combination and phase variation status of the haemoglobin receptors HmbR and HpuAB on meningococcal virulence. Microbiology (Reading) 157, 1446–1456. doi: 10.1099/mic.0.046946-0, PMID: 21310784 PMC3352162

[ref172] Taylor J. E. Swiderska A. Artero J. B. Callow P. Kneale G. (2012). Structural and functional analysis of the symmetrical Type I restriction endonuclease R.EcoR124I(NT). PLoS One 7, e35263–e35270. doi: 10.1371/journal.pone.0035263, PMID: 22493743 PMC3320862

[ref173] Taylor V. L. Titball R. W. Oyston P. C. F. (2005). Oral immunization with a dam mutant of Yersinia pseudotuberculosis protects against plague. Microbiology 151, 1919–1926. doi: 10.1099/mic.0.27959-0, PMID: 15941999

[ref174] Thomas C. B. Scavetta R. D. Gumport R. I. Churchill M. E. A. (2003). Structures of liganded and unliganded RsrI N6-adenine DNA methyltransferase: a distinct orientation for active cofactor binding. J. Biol. Chem. 278, 26094–26101. doi: 10.1074/jbc.M303751200, PMID: 12732637

[ref175] Tomcsanyi T. Berg D. E. (1989). Transposition effect of adenine (dam) methylation on activity of 0 end mutants of IS.50. J. Molo Biol 209, 191–193. doi: 10.1016/0022-2836(89)90271-4 2555517

[ref176] Torreblanca J. Casadesús J. (1996). DNA adenine Methylase mutants of salmonella Eylbhimurium and a novel dam-regulated locus. Genetics 144, 15–26. doi: 10.1093/genetics/144.1.15, PMID: 8878670 PMC1207489

[ref177] Tourancheau A. Mead E. A. Zhang X. Fang G. (2021). Discovering multiple types of DNA methylation from bacteria and microbiome using nanopore sequencing. Nat. Methods 18, 491–498. doi: 10.1038/s41592-021-01109-3, PMID: 33820988 PMC8107137

[ref178] Tran P. H. Korszun Z. R. Cerritelli S. Springhorn S. S. Lacks S. A. (1998). Crystal structure of the DpnM DNA adenine methyltransferase from the DpnII restriction system of Streptococcus pneumoniae bound to S-adenosylmethionine. Structure 6, 1563–1575. doi: 10.1016/S0969-2126(98)00154-3, PMID: 9862809

[ref179] Urig S. Gowher H. Hermann A. Beck C. Fatemi M. Humeny A.. (2002). The *Escherichia coli* dam DNA methyltransferase modifies DNA in a highly processive reaction. J. Mol. Biol. 319, 1085–1096. doi: 10.1016/S0022-2836(02)00371-6 12079349

[ref180] Val M. Kennedy S. P. Soler-Bistué A. J. Barbe V. Bouchier C. Ducos-Galand M.. (2014). Fuse or die: how to survive the loss of dam in vibrio cholerae. Mol. Microbiol. 91, 665–678. doi: 10.1111/mmi.12483, PMID: 24308271

[ref181] van der Woude M. W. Braaten B. A. Low D. A. (1992). Evidence for global regulatory control of pilus expression in *Escherichia coli* by Lrp and DNA methylation: model building based on analysis of pap. Mol. Microbiol. 6, 2429–2435. doi: 10.1111/j.1365-2958.1992.tb01418.x, PMID: 1357527

[ref182] Van der Woude M. Braaten B. Low D. (1996). Epigenetic phase variation of the pap operon in *Escherichia coli*. Trends Microbiol. 4, 5–9. doi: 10.1016/0966-842X(96)81498-3, PMID: 8824788

[ref183] van der Woude M. W. Henderson I. R. (2008). Regulation and function of Ag43 (flu). Annu. Rev. Microbiol. 62, 153–169. doi: 10.1146/annurev.micro.62.081307.162938, PMID: 18785838

[ref184] Vasu K. Nagaraja V. (2013). Diverse functions of restriction-modification systems in addition to cellular defense. Microbiol. Mol. Biol. Rev. 77, 53–72. doi: 10.1128/MMBR.00044-12, PMID: 23471617 PMC3591985

[ref185] Von Freiesleben U. Rasmussen K. V. Schaechter M. (1994). SeqA limits DnaA activity in replication from oriC in *Escherichia coli*. Mol. Microbiol. 14, 763–772. doi: 10.1111/j.1365-2958.1994.tb01313.x, PMID: 7891562

[ref186] Waldminghaus T. Skarstad K. (2009). The *Escherichia coli* SeqA protein. Plasmid 61, 141–150. doi: 10.1016/j.plasmid.2009.02.004, PMID: 19254745

[ref187] Watson M. E. Jr. Jarisch J. Smith A. L. (2004). Inactivation of deoxyadenosine methyltransferase (dam) attenuates *Haemophilus influenzae* virulence. Mol. Microbiol. 53, 651–664. doi: 10.1111/j.1365-2958.2004.04140.x, PMID: 15228541

[ref188] White-Ziegler C. A. Angus Hill M. L. Braaten B. A. van der Woude M. W. Low D. A. (1998). Thermoregulation of *Escherichia coli* pap transcription: H-NS is a temperature-dependent DNA methylation blocking factor. Mol. Microbiol. 28, 1121–1137. doi: 10.1046/j.1365-2958.1998.00872.x, PMID: 9680203

[ref189] White-Ziegler C. A. Black A. M. Eliades S. Young S. Porter K. (2002). The N-acetyltransferase RimJ responds to environmental stimuli to repress pap fimbrial transcription in *Escherichia coli*. J. Bacteriol. 184, 4334–4342. doi: 10.1128/JB.184.16.4334-4342.2002, PMID: 12142402 PMC135235

[ref190] Wion D. Casadesús J. (2006). N6-methyl-adenine: an epigenetic signal for DNA-protein interactions. Nat. Rev. Microbiol. 4, 183–192. doi: 10.1038/nrmicro1350, PMID: 16489347 PMC2755769

[ref191] Woodcock C. B. Horton J. R. Zhang X. Blumenthal R. M. Cheng X. (2020). Beta class amino methyltransferases from bacteria to humans: evolution and structural consequences. Nucleic Acids Res. 48, 10034–10044. doi: 10.1093/nar/gkaa446, PMID: 32453412 PMC7544214

[ref192] Woodcock C. B. Yakubov A. B. Reich N. O. (2017). *Caulobacter crescentus* cell cycle-regulated DNA Methyltransferase uses a novel mechanism for substrate recognition. Biochemistry 56, 3913–3922. doi: 10.1021/acs.biochem.7b00378, PMID: 28661661

[ref193] Yin H. Sun B. Zhou Y. Wang M. Xu Z. Fu Z.. (2014). A new strategy for methylated DNA detection based on photoelectrochemical immunosensor using Bi2S3 nanorods, methyl bonding domain protein and anti-his tag antibody. Biosens. Bioelectron. 51, 103–108. doi: 10.1016/j.bios.2013.07.040, PMID: 23948240

[ref194] Zaleski P. Piekarowicz A. (2004). Characterization of a dam mutant of Haemophilus influenzae Rd. Microbiology (Reading) 150, 3773–3781. doi: 10.1099/mic.0.27225-0, PMID: 15528663

[ref195] Zweiger G. Marczynski G. Shapiro L. (1994). A Caulobacter DNA Methyltransferase that functions only in the Predivisional cell. J. Mol. Biol. 235, 472–485. doi: 10.1006/jmbi.1994.1007, PMID: 8289276

